# Supplementary figures and images for: A comparative analysis of academic outcomes in blended versus traditional instructional approaches: An examination within the context of the National Medical Licensing Examination
Source: PLoS One. 2026 Apr 17;21(4):e0346793. doi: 10.1371/journal.pone.0346793 (PMC13089738; doi:10.1371/journal.pone.0346793)

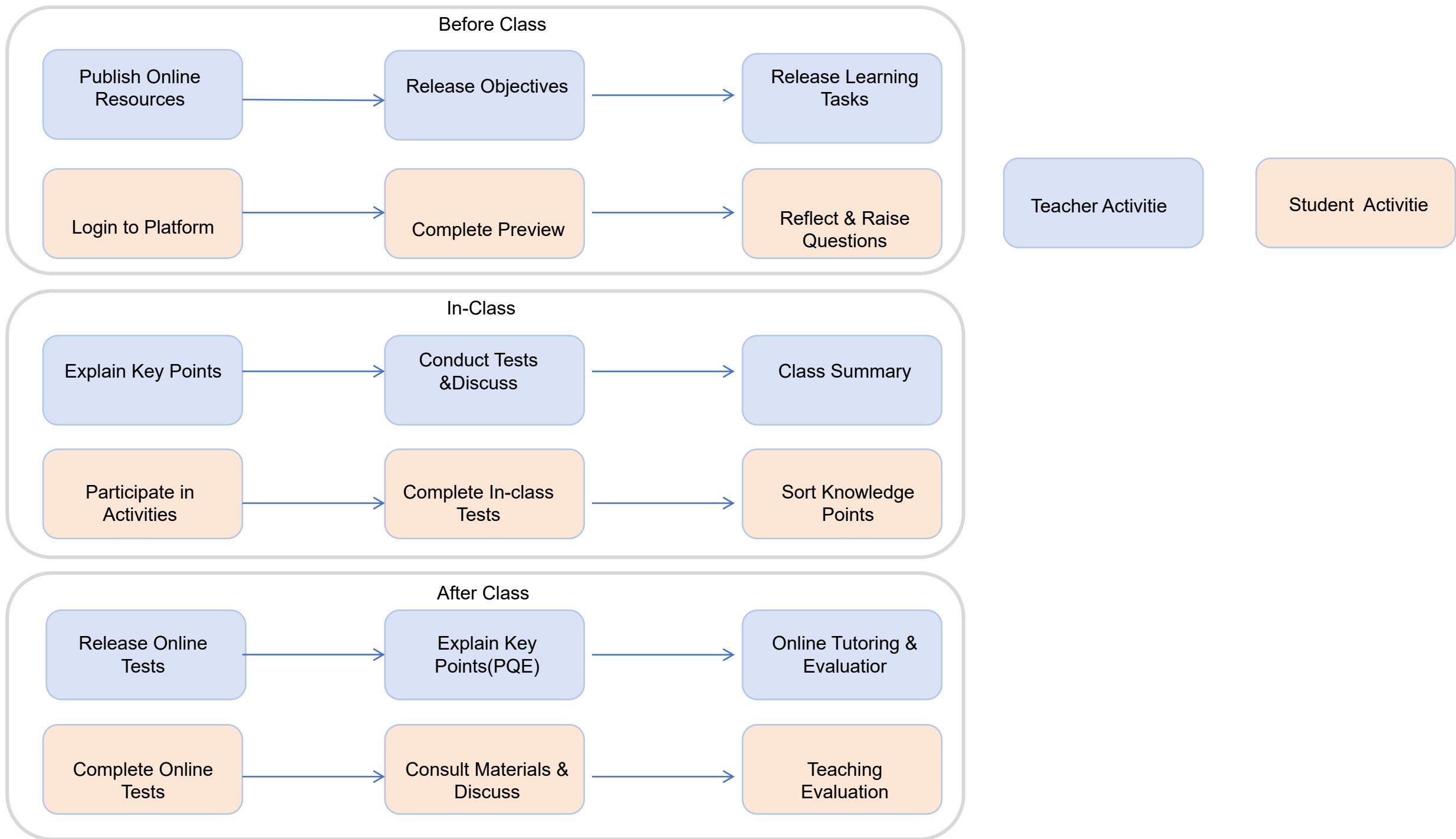

Supplement: S1 Fig — Schematic diagram of the blended teaching process integrating online and offline activities.The diagram illustrates the sequential teaching workflow across three phases: Before Class, In-Class, and After Class. Blue boxes represent teacher activities, while pink boxes represent corresponding student activities. The arrows indicate the progression of time and the interactive logic between teaching and learning steps. (PQE: Physician Qualification Examination) is the S1 Fig legend. (PDF) [file pone.0346793.s001.pdf]
