# Supplementary material for: A comparative analysis of academic outcomes in blended versus traditional instructional approaches: An examination within the context of the National Medical Licensing Examination
Source: PLoS One. 2026 Apr 17;21(4):e0346793. doi: 10.1371/journal.pone.0346793 (PMC13089738; doi:10.1371/journal.pone.0346793)
Supplement: S1 File — (PDF) [file pone.0346793.s002.pdf]

Funding Statement

This study was supported by 1. University-Level Teaching Reform Initiative at North Henan Medical University: An Empirical Investigation into the Blended Teaching Approach for Pathophysiology with a Focus on the Medical Licensing Examination (2023XJJG35). 2. "One Hospital, One Brand" Initiative by the Basic Medical College at North Henan Medical University: (yyyp2024001). 3. Henan Provincial Social Sciences Association: A Study on Developing High-Quality Faculty Teams in Medical Colleges Inspired by the Educators' Ethos (SKL-2024-805). 4. Outstanding Undergraduate Research Project Fund (2024002ZK, 2024004ZK, 2024008ZK, 2024031SK， 2026045ZK). 5. Fund for the Cultivation of Outstanding Young Teachers （SQ2022YQJH02）6.Henan Province University Student Innovation Training Project (S202513505014).7.Henan Province Science and Technology Innovation Project (262102310148).
