# Supplementary material for: A comparative analysis of academic outcomes in blended versus traditional instructional approaches: An examination within the context of the National Medical Licensing Examination
Source: PLoS One. 2026 Apr 17;21(4):e0346793. doi: 10.1371/journal.pone.0346793 (PMC13089738; doi:10.1371/journal.pone.0346793)
Supplement: S2 File — (PDF) [file pone.0346793.s003.pdf]

Information and informed consent in English

Information Sheet: Hello, students! We are from the Teaching and Research Section of Pathophysiology. We'd like to ask you a few questions, which will take about 1-3 minutes. This is to learn your evaluations and suggestions regarding the course, so as to provide guidance for the subsequent reform and optimization of the course.

Research Title: A Comparative Study of Blended and Traditional Teaching Achievements: Based on the Teaching of the National Medical Licensing Examination

Research Objectives: Currently, our teaching approaches incorporate both blended learning and traditional teaching. We aim to gain a more intuitive understanding of students' evaluations of blended learning and their overall situation. As blended learning is in its initial stage, this survey is essential to reflect its subsequent optimization needs and students' experiences. Additionally, the teaching and research group intends to enhance students' proficiency in the National Medical Licensing Examination through this research.

Research Benefits: Participation in this questionnaire does not provide direct benefits to participants. However, the research findings will be shared with all relevant stakeholders, including the school and the teaching and research team, to further optimize the curriculum. The outcomes of these optimizations will indirectly benefit participants by

enhancing the quality and effectiveness of the teaching.

Research Risks: This study poses no risks to participants and is solely for reference in teaching reform.

Participants' Rights: Participation in this study is entirely voluntary, and participants have the right to withdraw at any time. You may skip questions you do not wish to answer and ask for clarification on any questions you do not understand at any point.

Confidentiality: This questionnaire does not require your name. All information provided will be kept strictly confidential.

ENGLISH QUESTIONNAIRE

1. How much did you know about the blended teaching model before learning Pathophysiology?	A. Have no knowledge at all<br>B. Know a little<br>C. Know fairly well<br>D. Know a great deal
2. What do you think is the impact of the blended teaching model of Pathophysiology on your learning effect?	A. Has no impact<br>B. Has a certain impact<br>C. Has a relatively large impact<br>D. Has a significant impact<br>E. Black
3. So far, have you adapted to the classroom model of blended teaching in Pathophysiology?	A. Completely adapted<br>B. Adapted<br>C. Somewhat unadapted<br>D. Unadapted<br>E. Black
4. Which teaching resources do you prefer to use in the study of Pathophysiology?	A. Textbook<br>B. Videos on Bilibili and other platforms<br>C. Resources pushed by Xuetangyun<br>D. Learning resources of Youmu courses<br>E. Others (please specify)
5. After completing the learning resources pushed by Xuetangyun (online learning platform), can you independently finish the	A. Able to<br>B. Unable to<br>C. Unsure

practice questions in the courseware?	
6. Do you think you have a clear understanding of the content related to Pathophysiology in the National Medical Licensing Examination syllabus so far?	A. Very clear<br>B. Clear<br>C. Roughly clear<br>D. Unclear
7. Do you think the study of Pathophysiology helps you understand relevant clinical knowledge?	A. Very helpful<br>B. Helpful<br>C. Not very helpful<br>D. Not helpful
8. What capabilities do you think blended teaching has improved for you?	A. Self - learning ability<br>B. Communication and expression ability<br>C. Independent thinking ability<br>D. Other abilities [Please specify]<br>E. No improvement
9. Are you satisfied with the teaching of Pathophysiology?	A. Very satisfied<br>B. Satisfied<br>C. Basically satisfied<br>D. Dissatisfied
