# Supplementary material for: A comparative analysis of academic outcomes in blended versus traditional instructional approaches: An examination within the context of the National Medical Licensing Examination
Source: PLoS One. 2026 Apr 17;21(4):e0346793. doi: 10.1371/journal.pone.0346793 (PMC13089738; doi:10.1371/journal.pone.0346793)
Supplement: S3 File — (PDF) [file pone.0346793.s004.pdf]

Pathophysiology

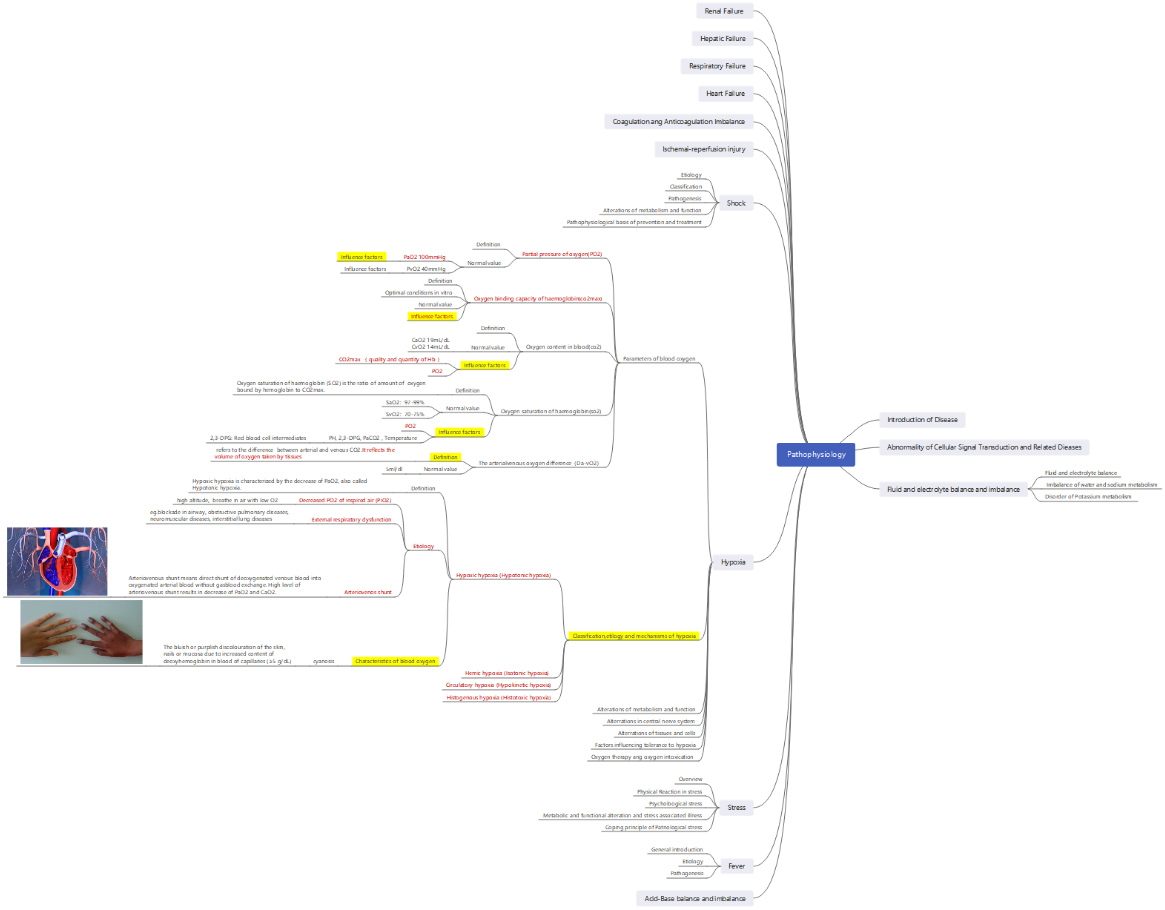

1. Introduction of Disease

1.1. Concept of Diease

1.2. Etiology of Diease

1.3. Pathogenesis of Diease

1.4. Outcome of Diease

2. Abnormality of Cellular Signal Transduction and Related Dieases

2.1. Overview of Cell Signal

2.2. Mechanisms of Abnormal Cell Signal Transduction

2.3. Cell Signal Transduction Abnormality and DIease

2.4. Pathophysiologocal Basis of Prevevtion and Treatment

3. Fluid and electrolyte balance and imbalance

3.1. Fluid and electrolyte balance

3.2. Imbalance of water and sodium metabolism

3.3. Disorder of Potassium metabolism

4. Acid-Base balance and imbalance

4.1. Normal Acid-base balance

4.2. Parameters of Acid-base balance

4.3. Simple Acid-base disturbance

4.4. Mixed Acid-base disturbance

4.5. Guidelines for the diagnosis of acid-base disturbances

5. Fever

5.1. General introduction

5.2. Etiology

5.3. Pathogenesis

6. Stress

6.1. Overview

6.2. Physical Reaction in stress

6.3. Psycholoogical stress

6.4. Metabolic and functional alteration and stress associated illness

6.5. Coping principle of Patnological stress

7. Hypoxia

7.1. Parameters of blood oxygen

7.1.1. Partial pressure of oxygen(PO2)

7.1.1.1. Definition

7.1.1.1.1. Partial pressure of oxygen(PO2) is in blood is the portion of total blood gas pressure exerted by oxygen physically dissolved in blood.

7.1.1.2. Normal value

7.1.1.2.1. PaO2 100mmHg

7.1.1.2.1.1. Influence factors

PO2 of inspired air(PiO2)

pulmonary function

7.1.1.2.2. PvO2 40mmHg

7.1.1.2.2.1. Influence factors

internal respiration

7.1.2. Oxygen binding capacity of haemoglobin(co2max)

7.1.2.1. Definition

7.1.2.1.1. xygen binding capacity of blood (CO2max) is the maximal volume of oxygen that can be bound to hemoglobin (Hb) in 100 mL blood. （Physical dissolve+Hb carries oxygen）

7.1.2.2. Optimal conditions in vitro

7.1.2.2.1. PO_2 ： 150 mmHg PCO_2 ： 40 mmHg
Temperature:38□

7.1.2.3. Normal value

7.1.2.3.1. Physical dissolve：0.31 ml/dl Hb carries oxygen：1.34mL/g x 15g/dL = 20mL/dL CO_2max = Hb
carries oxygen

7.1.2.4. Influence factors

7.1.2.4.1. quality (affinity for O_2) and quantity of Hb

7.1.3. Oxygen content in blood(co2)

7.1.3.1. Definition

7.1.3.1.1. Oxygen content in blood (CO2) ：The actual content of oxygen in 100 mL blood. (Physical dissolve+Hb carries oxygen)

7.1.3.2. Normal value

7.1.3.2.1. CaO_2 19mL/dL CvO_2 14mL/dL

7.1.3.3. Influence factors

7.1.3.3.1. CO_2max （ quality and quantity of Hb ）

7.1.3.3.2. PO_2

7.1.4. Oxygen saturation of haemoglobin(so2)

7.1.4.1. Definition

7.1.4.1.1. Oxygen saturation of haemoglobin (SO_2) is the ratio of amount of oxygen bound by hemoglobin to CO_2max.

7.1.4.2. Normal value

7.1.4.2.1. SaO_2：97-99％

7.1.4.2.2. SvO_2：70-75％

7.1.4.3. Influence factors

7.1.4.3.1. PO_2

7.1.4.3.2. PH, 2,3-DPG, $PaCO_2$, Temperature

7.1.4.3.2.1. 2,3-DPG: Red blood cell intermediates

7.1.5. The arterialvenous oxygen difference（Da-vO2）

7.1.5.1. Definition

7.1.5.1.1. refers to the difference between arterial and venous CO2.It reflects the volume of oxygen taken by tissues

7.1.5.2. Normal value

7.1.5.2.1. 5ml/dl

7.2. Classification,etilogy and mechanisms of hypoxia

7.2.1. Hypoxic hypoxia (Hypotonic hypoxia)

7.2.1.1. Definition

7.2.1.1.1. Hypoxic hypoxia is characterized by the decrease of PaO2, also called Hypotonic hypoxia.

7.2.1.2. Etiology

7.2.1.2.1. Decreased PO2 of inspired air (PiO2)

7.2.1.2.1.1. high altitude, breathe in air with low O2

7.2.1.2.2. External respiratory dysfunction

7.2.1.2.2.1. eg.blockade in airway, obstructive pulmonary diseases, neuromuscular diseases, interstitial lung diseases

7.2.1.2.3. Arteriovenos shunt

7.2.1.2.3.1. Arteriovenous shunt means direct shunt of deoxygenated venous blood into oxygenated arterial blood without gasblood exchange. High level of arteriovenous shunt results in decrease of PaO2 and CaO2.

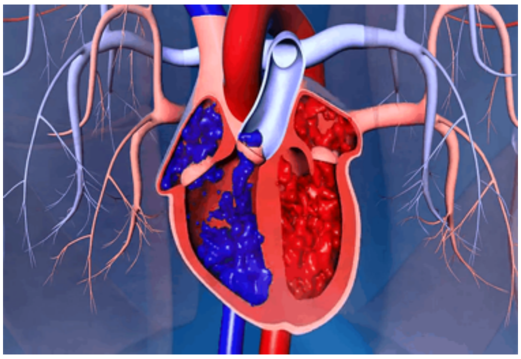

7.2.1.3. Characteristics of blood oxygen

7.2.1.3.1. cyanosis

7.2.1.3.1.1. The bluish or purplish discolouration of the skin, nails or mucosa due to increased content of deoxyhemoglobin in blood of capillaries (≥5 g/dL)

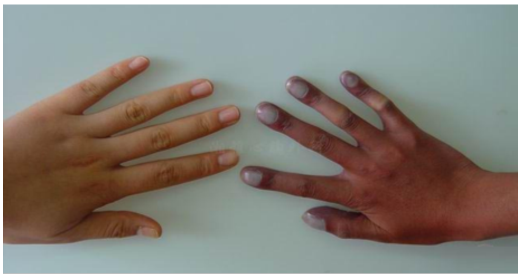

7.2.2. Hemic hypoxia (Isotonic hypoxia)

7.2.3. Circulatory hypoxia (Hypokinetic hypoxia)

7.2.4. Histogenous hypoxia (Histotoxic hypoxia)

7.3. Alterations of metabolism and function

7.4. Alterrations in central nerve system

7.5. Alterrations of tissues and cells

7.6. Factors influencing tolerance to hypoxia

7.7. Oxygen therapy ang oxygen intoxication

8. Shock

8.1. Etiology

8.2. Classification

8.3. Pathogenesis

8.4. Alterations of metabolism and function

8.5. Pathophysiological basis of prevention and treatment

9. Ischemai-reperfusion injury

10. Coagulation ang Anticoagulation Imbalance

11. Heart Failure

12. Respiratory Failure

13. Hepatic Failure

14. Renal Failure
