# Supplementary material for: A comparative analysis of academic outcomes in blended versus traditional instructional approaches: An examination within the context of the National Medical Licensing Examination
Source: PLoS One. 2026 Apr 17;21(4):e0346793. doi: 10.1371/journal.pone.0346793 (PMC13089738; doi:10.1371/journal.pone.0346793)
Supplement: S4 File — Midterm Scores, Final Scores, and Average Scores are the S4 File legend. (PDF) [file pone.0346793.s005.pdf]

Explain：The minimal data set consists of the grades of each class. It has been attached at the end.Cohen's d and the 95% confidence interval were calculated using Python.

Average exam scores of the experimental group and the control group during the mid-term examination									
the experimental group	mean score	SD	the control group	mean score	SD	t	p	Cohen's d	95% CI
1-4<br>9-12<br>17-19<br>117-119	66<br>64<br>68<br>67	1.708	5-8<br>13-16<br>20-22<br>120-122	62<br>61<br>62<br>66	2.217	2.501	0.047	2.01	0.218,3.806
all the experimental group	66.25		all the control group	62.75					

Average final exam scores of the experimental group and the control group									
the experimental group	mean score	SD	the control group	mean score	SD	t	p	Cohen's d	95% CI
1-4<br>9-12<br>17-19<br>117-119	77<br>76<br>76<br>78	1.155	5-8<br>13-16<br>20-22<br>120-122	75<br>74<br>73<br>75	0.96	3.667	0.011	2.894	0.821, 4.973
All the experimental group	76.75		All the control group	74.25					

1-4　Mid-term performance			
curriculum code	course	class	report card
0111117	Pathophysiology	1	82
0111117	Pathophysiology	1	58.25
0111117	Pathophysiology	1	89.5
0111117	Pathophysiology	1	74.5
0111117	Pathophysiology	1	47
0111117	Pathophysiology	1	84.5
0111117	Pathophysiology	1	87
0111117	Pathophysiology	1	55.75
0111117	Pathophysiology	1	82
0111117	Pathophysiology	1	88.25
0111117	Pathophysiology	1	74.5
0111117	Pathophysiology	1	88.25
0111117	Pathophysiology	1	45.75
0111117	Pathophysiology	1	54.5
0111117	Pathophysiology	1	52
0111117	Pathophysiology	1	70.75
0111117	Pathophysiology	1	88.25
0111117	Pathophysiology	1	47
0111117	Pathophysiology	1	53.25
0111117	Pathophysiology	1	53.25
0111117	Pathophysiology	1	59.5
0111117	Pathophysiology	1	60.75
0111117	Pathophysiology	1	68.25
0111117	Pathophysiology	1	42
0111117	Pathophysiology	1	52
0111117	Pathophysiology	1	89.5
0111117	Pathophysiology	1	55.75
0111117	Pathophysiology	1	67
0111117	Pathophysiology	1	59.5
0111117	Pathophysiology	1	53.25
0111117	Pathophysiology	2	83.25
0111117	Pathophysiology	2	95.75
0111117	Pathophysiology	2	78.25
0111117	Pathophysiology	2	64.5
0111117	Pathophysiology	2	83.25
0111117	Pathophysiology	2	70.75
0111117	Pathophysiology	2	60.75
0111117	Pathophysiology	2	59.5
0111117	Pathophysiology	2	55.75
0111117	Pathophysiology	2	72
0111117	Pathophysiology	2	43.25

0111117	Pathophysiology	2	43.25
0111117	Pathophysiology	2	58.25
0111117	Pathophysiology	2	89.5
0111117	Pathophysiology	2	72
0111117	Pathophysiology	2	67
0111117	Pathophysiology	2	53.25
0111117	Pathophysiology	2	63.25
0111117	Pathophysiology	2	54.5
0111117	Pathophysiology	2	53.25
0111117	Pathophysiology	2	64.5
0111117	Pathophysiology	2	75.75
0111117	Pathophysiology	2	49.5
0111117	Pathophysiology	2	84.5
0111117	Pathophysiology	2	78.25
0111117	Pathophysiology	2	67
0111117	Pathophysiology	2	53.25
0111117	Pathophysiology	2	49.5
0111117	Pathophysiology	2	77
0111117	Pathophysiology	2	44.5
0111117	Pathophysiology	3	59.5
0111117	Pathophysiology	3	68.25
0111117	Pathophysiology	3	68.25
0111117	Pathophysiology	3	82
0111117	Pathophysiology	3	97
0111117	Pathophysiology	3	77
0111117	Pathophysiology	3	42
0111117	Pathophysiology	3	80.75
0111117	Pathophysiology	3	48.25
0111117	Pathophysiology	3	88.25
0111117	Pathophysiology	3	88.25
0111117	Pathophysiology	3	68.25
0111117	Pathophysiology	3	50.75
0111117	Pathophysiology	3	53.25
0111117	Pathophysiology	3	72
0111117	Pathophysiology	3	48.25
0111117	Pathophysiology	3	62
0111117	Pathophysiology	3	57
0111117	Pathophysiology	3	53.25
0111117	Pathophysiology	3	52
0111117	Pathophysiology	3	63.25
0111117	Pathophysiology	3	73.25
0111117	Pathophysiology	3	43.25
0111117	Pathophysiology	3	57

0111117	Pathophysiology	3	77
0111117	Pathophysiology	3	82
0111117	Pathophysiology	3	53.25
0111117	Pathophysiology	3	44.5
0111117	Pathophysiology	3	64.5
0111117	Pathophysiology	3	59.5
0111117	Pathophysiology	4	80.75
0111117	Pathophysiology	4	88.25
0111117	Pathophysiology	4	69.5
0111117	Pathophysiology	4	63.25
0111117	Pathophysiology	4	69.5
0111117	Pathophysiology	4	75.75
0111117	Pathophysiology	4	58.25
0111117	Pathophysiology	4	74.5
0111117	Pathophysiology	4	83.25
0111117	Pathophysiology	4	75.75
0111117	Pathophysiology	4	59.5
0111117	Pathophysiology	4	78.25
0111117	Pathophysiology	4	70.75
0111117	Pathophysiology	4	73.25
0111117	Pathophysiology	4	92
0111117	Pathophysiology	4	60.75
0111117	Pathophysiology	4	47
0111117	Pathophysiology	4	62
0111117	Pathophysiology	4	54.5
0111117	Pathophysiology	4	65.75
0111117	Pathophysiology	4	68.25
0111117	Pathophysiology	4	73.25
0111117	Pathophysiology	4	74.5
0111117	Pathophysiology	4	52
0111117	Pathophysiology	4	55.75
0111117	Pathophysiology	4	48.25
0111117	Pathophysiology	4	44.5
0111117	Pathophysiology	4	75.75
0111117	Pathophysiology	4	68.25
0111117	Pathophysiology	4	57
0111117	Pathophysiology	4	77
0111117	Pathophysiology	4	47
mean score			65.79098361

5-8 Mid-term performance			
curriculum code	course	class	report card
0111117	Pathophysiology	5	70.75
0111117	Pathophysiology	5	85.75
0111117	Pathophysiology	5	78.25
0111117	Pathophysiology	5	48.25
0111117	Pathophysiology	5	39.5
0111117	Pathophysiology	5	89.5
0111117	Pathophysiology	5	42
0111117	Pathophysiology	5	47
0111117	Pathophysiology	5	52
0111117	Pathophysiology	5	54.5
0111117	Pathophysiology	5	78.25
0111117	Pathophysiology	5	78.25
0111117	Pathophysiology	5	64.5
0111117	Pathophysiology	5	53.25
0111117	Pathophysiology	5	75.75
0111117	Pathophysiology	5	52
0111117	Pathophysiology	5	42
0111117	Pathophysiology	5	74.5
0111117	Pathophysiology	5	43.25
0111117	Pathophysiology	5	69.5
0111117	Pathophysiology	5	72
0111117	Pathophysiology	5	42
0111117	Pathophysiology	5	79.5
0111117	Pathophysiology	5	74.5
0111117	Pathophysiology	5	58.25
0111117	Pathophysiology	5	44.5
0111117	Pathophysiology	5	74.5
0111117	Pathophysiology	5	55.75
0111117	Pathophysiology	5	43.25
0111117	Pathophysiology	5	88.25
0111117	Pathophysiology	5	60.75
0111117	Pathophysiology	6	93.25
0111117	Pathophysiology	6	82
0111117	Pathophysiology	6	79.5
0111117	Pathophysiology	6	65.75
0111117	Pathophysiology	6	53.25
0111117	Pathophysiology	6	87
0111117	Pathophysiology	6	45.75
0111117	Pathophysiology	6	65.75
0111117	Pathophysiology	6	60.75
0111117	Pathophysiology	6	42

0111117	Pathophysiology	6	45.75
0111117	Pathophysiology	6	67
0111117	Pathophysiology	6	40.75
0111117	Pathophysiology	6	69.5
0111117	Pathophysiology	6	54.5
0111117	Pathophysiology	6	40.75
0111117	Pathophysiology	6	50.75
0111117	Pathophysiology	6	55.75
0111117	Pathophysiology	6	53.25
0111117	Pathophysiology	6	68.25
0111117	Pathophysiology	6	65.75
0111117	Pathophysiology	6	58.25
0111117	Pathophysiology	6	45.75
0111117	Pathophysiology	6	38.25
0111117	Pathophysiology	6	33.25
0111117	Pathophysiology	6	65.75
0111117	Pathophysiology	6	48.25
0111117	Pathophysiology	6	59.5
0111117	Pathophysiology	6	65.75
0111117	Pathophysiology	7	57
0111117	Pathophysiology	7	60.75
0111117	Pathophysiology	7	87
0111117	Pathophysiology	7	74.5
0111117	Pathophysiology	7	43.25
0111117	Pathophysiology	7	69.5
0111117	Pathophysiology	7	87
0111117	Pathophysiology	7	43.25
0111117	Pathophysiology	7	40.75
0111117	Pathophysiology	7	55.75
0111117	Pathophysiology	7	60.75
0111117	Pathophysiology	7	53.25
0111117	Pathophysiology	7	63.25
0111117	Pathophysiology	7	37
0111117	Pathophysiology	7	79.5
0111117	Pathophysiology	7	52
0111117	Pathophysiology	7	79.5
0111117	Pathophysiology	7	77
0111117	Pathophysiology	7	64.5
0111117	Pathophysiology	7	60.75
0111117	Pathophysiology	7	47
0111117	Pathophysiology	7	82
0111117	Pathophysiology	7	69.5
0111117	Pathophysiology	7	50.75

0111117	Pathophysiology	7	72
0111117	Pathophysiology	7	77
0111117	Pathophysiology	7	49.5
0111117	Pathophysiology	7	54.5
0111117	Pathophysiology	7	79.5
0111117	Pathophysiology	7	52
0111117	Pathophysiology	8	77
0111117	Pathophysiology	8	87
0111117	Pathophysiology	8	77
0111117	Pathophysiology	8	88.25
0111117	Pathophysiology	8	90.75
0111117	Pathophysiology	8	79.5
0111117	Pathophysiology	8	65.75
0111117	Pathophysiology	8	43.25
0111117	Pathophysiology	8	48.25
0111117	Pathophysiology	8	63.25
0111117	Pathophysiology	8	37
0111117	Pathophysiology	8	55.75
0111117	Pathophysiology	8	67
0111117	Pathophysiology	8	80.75
0111117	Pathophysiology	8	84.5
0111117	Pathophysiology	8	65.75
0111117	Pathophysiology	8	80.75
0111117	Pathophysiology	8	63.25
0111117	Pathophysiology	8	62
0111117	Pathophysiology	8	63.25
0111117	Pathophysiology	8	43.25
0111117	Pathophysiology	8	42
0111117	Pathophysiology	8	57
0111117	Pathophysiology	8	37
0111117	Pathophysiology	8	35.75
0111117	Pathophysiology	8	49.5
0111117	Pathophysiology	8	79.5
0111117	Pathophysiology	8	38.25
0111117	Pathophysiology	8	53.25
mean score			61.59033613

9-12 Mid-term performance				
curriculum code	course	class	report card	Special Circumstances Explanation
0111117	Pathophysiology	9	73.25	
0111117	Pathophysiology	9	68.25	
0111117	Pathophysiology	9	74.5	
0111117	Pathophysiology	9	64.5	
0111117	Pathophysiology	9	88.25	
0111117	Pathophysiology	9	83.25	
0111117	Pathophysiology	9	82	
0111117	Pathophysiology	9	83.25	
0111117	Pathophysiology	9	72	
0111117	Pathophysiology	9	65.75	
0111117	Pathophysiology	9	83.25	
0111117	Pathophysiology	9	70.75	
0111117	Pathophysiology	9	68.25	
0111117	Pathophysiology	9	60.75	
0111117	Pathophysiology	9	74.5	
0111117	Pathophysiology	9	73.25	
0111117	Pathophysiology	9	74.5	
0111117	Pathophysiology	9	52	
0111117	Pathophysiology	9	74.5	
0111117	Pathophysiology	9	72	
0111117	Pathophysiology	9	87	
0111117	Pathophysiology	9	82	
0111117	Pathophysiology	9	84.5	
0111117	Pathophysiology	9	94.5	
0111117	Pathophysiology	9	83.25	
0111117	Pathophysiology	9	65.75	
0111117	Pathophysiology	9	58.25	
0111117	Pathophysiology	9	57	
0111117	Pathophysiology	9	40.75	
0111117	Pathophysiology	9	37	
0111117	Pathophysiology	9	72	
0111117	Pathophysiology	9	70.75	
0111117	Pathophysiology	9	64.5	
0111117	Pathophysiology	9	59.5	
0111117	Pathophysiology	10	53.25	
0111117	Pathophysiology	10	70.75	
0111117	Pathophysiology	10	83.25	
0111117	Pathophysiology	10	63.25	
0111117	Pathophysiology	10	77	
0111117	Pathophysiology	10	63.25	

0111117	Pathophysiology	10	74.5	
0111117	Pathophysiology	10	49.5	
0111117	Pathophysiology	10	53.25	
0111117	Pathophysiology	10	50.75	
0111117	Pathophysiology	10	40.75	
0111117	Pathophysiology	10	68.25	
0111117	Pathophysiology	10	78.25	
0111117	Pathophysiology	10	54.5	
0111117	Pathophysiology	10	0	
0111117	Pathophysiology	10	77	
0111117	Pathophysiology	10	70.75	
0111117	Pathophysiology	10	88.25	
0111117	Pathophysiology	10	59.5	
0111117	Pathophysiology	10	37	
0111117	Pathophysiology	10	44.5	
0111117	Pathophysiology	10	49.5	
0111117	Pathophysiology	10	43.25	
0111117	Pathophysiology	10	62	
0111117	Pathophysiology	10	43.25	
0111117	Pathophysiology	10	67	
0111117	Pathophysiology	10	55.75	
0111117	Pathophysiology	10	44.5	
0111117	Pathophysiology	10	68.25	
0111117	Pathophysiology	10	65.75	
0111117	Pathophysiology	10	33.25	
0111117	Pathophysiology	11	73.25	
0111117	Pathophysiology	11	77	
0111117	Pathophysiology	11	85.75	
0111117	Pathophysiology	11	75.75	
0111117	Pathophysiology	11	59.5	
0111117	Pathophysiology	11	82	
0111117	Pathophysiology	11	93.25	
0111117	Pathophysiology	11	80.75	
0111117	Pathophysiology	11	74.5	
0111117	Pathophysiology	11	87	
0111117	Pathophysiology	11	44.5	
0111117	Pathophysiology	11	54.5	
0111117	Pathophysiology	11	47	
0111117	Pathophysiology	11	25.75	
0111117	Pathophysiology	11	55.75	
0111117	Pathophysiology	11	42	
0111117	Pathophysiology	11	83.25	
0111117	Pathophysiology	11	60.75	

0111117	Pathophysiology	11	59.5	
0111117	Pathophysiology	11	37	
0111117	Pathophysiology	11	89.5	
0111117	Pathophysiology	11	63.25	
0111117	Pathophysiology	11	63.25	
0111117	Pathophysiology	11	82	
0111117	Pathophysiology	11	68.25	
0111117	Pathophysiology	11	59.5	
0111117	Pathophysiology	11	80.75	
0111117	Pathophysiology	11	53.25	
0111117	Pathophysiology	11	54.5	
0111117	Pathophysiology	11	57	
0111117	Pathophysiology	11	78.25	
0111117	Pathophysiology	11	65.75	
0111117	Pathophysiology	12	65.75	
0111117	Pathophysiology	12	55.75	
0111117	Pathophysiology	12	55.75	
0111117	Pathophysiology	12	87	
0111117	Pathophysiology	12	73.25	
0111117	Pathophysiology	12	52	
0111117	Pathophysiology	12	54.5	
0111117	Pathophysiology	12	54.5	
0111117	Pathophysiology	12	79.5	
0111117	Pathophysiology	12	63.25	
0111117	Pathophysiology	12	75.75	
0111117	Pathophysiology	12	58.25	
0111117	Pathophysiology	12	35.75	
0111117	Pathophysiology	12	58.25	
0111117	Pathophysiology	12	52	
0111117	Pathophysiology	12	72	
0111117	Pathophysiology	12	57	
0111117	Pathophysiology	12	47	
0111117	Pathophysiology	12	50.75	
0111117	Pathophysiology	12	59.5	
0111117	Pathophysiology	12	60.75	
0111117	Pathophysiology	12	70.75	
0111117	Pathophysiology	12	50.75	
0111117	Pathophysiology	12	57	
0111117	Pathophysiology	12	68.25	
0111117	Pathophysiology	12	70.75	
0111117	Pathophysiology	12	42	
0111117	Pathophysiology	12	49.5	
0111117	Pathophysiology	12	47	

0111117	Pathophysiology	12	0	Absence from the exam will not be included in the average score.
mean score			63.83928571	

13-16 Mid-term performance			
curriculum code	course	class	report card
0111117	Pathophysiology	13	38.25
0111117	Pathophysiology	13	55.75
0111117	Pathophysiology	13	78.25
0111117	Pathophysiology	13	65.75
0111117	Pathophysiology	13	73.25
0111117	Pathophysiology	13	74.5
0111117	Pathophysiology	13	69.5
0111117	Pathophysiology	13	73.25
0111117	Pathophysiology	13	67
0111117	Pathophysiology	13	0
0111117	Pathophysiology	13	92
0111117	Pathophysiology	13	90.75
0111117	Pathophysiology	13	72
0111117	Pathophysiology	13	79.5
0111117	Pathophysiology	13	75.75
0111117	Pathophysiology	13	52
0111117	Pathophysiology	13	52
0111117	Pathophysiology	13	72
0111117	Pathophysiology	13	57
0111117	Pathophysiology	13	68.25
0111117	Pathophysiology	13	70.75
0111117	Pathophysiology	13	82
0111117	Pathophysiology	13	79.5
0111117	Pathophysiology	13	52
0111117	Pathophysiology	13	65.75
0111117	Pathophysiology	13	79.5
0111117	Pathophysiology	13	65.75
0111117	Pathophysiology	13	35.75
0111117	Pathophysiology	13	65.5
0111117	Pathophysiology	13	79.5
0111117	Pathophysiology	13	30.75
0111117	Pathophysiology	14	98.25
0111117	Pathophysiology	14	89.5
0111117	Pathophysiology	14	73.25

0111117	Pathophysiology	14	42
0111117	Pathophysiology	14	62
0111117	Pathophysiology	14	78.25
0111117	Pathophysiology	14	63.25
0111117	Pathophysiology	14	60.75
0111117	Pathophysiology	14	69.5
0111117	Pathophysiology	14	79.5
0111117	Pathophysiology	14	83.25
0111117	Pathophysiology	14	58.25
0111117	Pathophysiology	14	85.75
0111117	Pathophysiology	14	79.5
0111117	Pathophysiology	14	72
0111117	Pathophysiology	14	55.75
0111117	Pathophysiology	14	62
0111117	Pathophysiology	14	53.25
0111117	Pathophysiology	14	72
0111117	Pathophysiology	14	50.75
0111117	Pathophysiology	14	47
0111117	Pathophysiology	14	50.75
0111117	Pathophysiology	14	59.5
0111117	Pathophysiology	14	55.75
0111117	Pathophysiology	14	47
0111117	Pathophysiology	14	64.5
0111117	Pathophysiology	14	67
0111117	Pathophysiology	14	43.25
0111117	Pathophysiology	14	45.75
0111117	Pathophysiology	14	59.5
0111117	Pathophysiology	14	60.75
0111117	Pathophysiology	15	74.5
0111117	Pathophysiology	15	85.75
0111117	Pathophysiology	15	52
0111117	Pathophysiology	15	55.75
0111117	Pathophysiology	15	44.5
0111117	Pathophysiology	15	62
0111117	Pathophysiology	15	78.25
0111117	Pathophysiology	15	60.75
0111117	Pathophysiology	15	78.25
0111117	Pathophysiology	15	58.25
0111117	Pathophysiology	15	72
0111117	Pathophysiology	15	83.25
0111117	Pathophysiology	15	53.25
0111117	Pathophysiology	15	85.75
0111117	Pathophysiology	15	38.25

0111117	Pathophysiology	15	88.25
0111117	Pathophysiology	15	49.5
0111117	Pathophysiology	15	45.75
0111117	Pathophysiology	15	52
0111117	Pathophysiology	15	49.5
0111117	Pathophysiology	15	40.75
0111117	Pathophysiology	15	40.75
0111117	Pathophysiology	15	43.25
0111117	Pathophysiology	15	42
0111117	Pathophysiology	15	32
0111117	Pathophysiology	15	40.75
0111117	Pathophysiology	15	67
0111117	Pathophysiology	15	50.75
0111117	Pathophysiology	15	55.75
0111117	Pathophysiology	15	55.75
0111117	Pathophysiology	15	67
0111117	Pathophysiology	16	95.75
0111117	Pathophysiology	16	67
0111117	Pathophysiology	16	40.75
0111117	Pathophysiology	16	74.5
0111117	Pathophysiology	16	49.5
0111117	Pathophysiology	16	80.75
0111117	Pathophysiology	16	53.25
0111117	Pathophysiology	16	67
0111117	Pathophysiology	16	53.25
0111117	Pathophysiology	16	65.75
0111117	Pathophysiology	16	33.25
0111117	Pathophysiology	16	87
0111117	Pathophysiology	16	34.5
0111117	Pathophysiology	16	74.5
0111117	Pathophysiology	16	60.75
0111117	Pathophysiology	16	45.75
0111117	Pathophysiology	16	55.75
0111117	Pathophysiology	16	82
0111117	Pathophysiology	16	59.5
0111117	Pathophysiology	16	43.25
0111117	Pathophysiology	16	57
0111117	Pathophysiology	16	50.75
0111117	Pathophysiology	16	43.25
0111117	Pathophysiology	16	43.25
0111117	Pathophysiology	16	54.5
0111117	Pathophysiology	16	52
0111117	Pathophysiology	16	52

0111117	Pathophysiology	16	85.75
0111117	Pathophysiology	16	50.75
0111117	Pathophysiology	16	50.75
0111117	Pathophysiology	16	52
0111117	Pathophysiology	16	54.5
mean score			61.412

117-119 Mid-term performance			
curriculum code	course	class	report card
0111117	Pathophysiology	117	63.25
0111117	Pathophysiology	117	52
0111117	Pathophysiology	117	62
0111117	Pathophysiology	117	67
0111117	Pathophysiology	117	64.5
0111117	Pathophysiology	117	78.25
0111117	Pathophysiology	117	79.5
0111117	Pathophysiology	117	74.5
0111117	Pathophysiology	117	53.25
0111117	Pathophysiology	117	48.25
0111117	Pathophysiology	117	68.25
0111117	Pathophysiology	117	60.75
0111117	Pathophysiology	117	75.75
0111117	Pathophysiology	117	53.25
0111117	Pathophysiology	117	74.5
0111117	Pathophysiology	117	78.25
0111117	Pathophysiology	117	47
0111117	Pathophysiology	117	77
0111117	Pathophysiology	117	55.75
0111117	Pathophysiology	117	82
0111117	Pathophysiology	117	62
0111117	Pathophysiology	117	62
0111117	Pathophysiology	117	72
0111117	Pathophysiology	117	63.25
0111117	Pathophysiology	117	75.75
0111117	Pathophysiology	117	50.75
0111117	Pathophysiology	117	65.75
0111117	Pathophysiology	117	65.75
0111117	Pathophysiology	117	65.75
0111117	Pathophysiology	117	82

0111117	Pathophysiology	118	67
0111117	Pathophysiology	118	65.75
0111117	Pathophysiology	118	70.75
0111117	Pathophysiology	118	77
0111117	Pathophysiology	118	84.5
0111117	Pathophysiology	118	77
0111117	Pathophysiology	118	48.25
0111117	Pathophysiology	118	89.5
0111117	Pathophysiology	118	80.75
0111117	Pathophysiology	118	43.25
0111117	Pathophysiology	118	80.75
0111117	Pathophysiology	118	59.5
0111117	Pathophysiology	118	68.25
0111117	Pathophysiology	118	64.5
0111117	Pathophysiology	118	59.5
0111117	Pathophysiology	118	87
0111117	Pathophysiology	118	50.75
0111117	Pathophysiology	118	39.5
0111117	Pathophysiology	118	62
0111117	Pathophysiology	118	67
0111117	Pathophysiology	118	33.25
0111117	Pathophysiology	118	67
0111117	Pathophysiology	118	77
0111117	Pathophysiology	118	73.25
0111117	Pathophysiology	118	47
0111117	Pathophysiology	118	84.5
0111117	Pathophysiology	118	95.75
0111117	Pathophysiology	118	78.25
0111117	Pathophysiology	118	83.25
0111117	Pathophysiology	118	67
0111117	Pathophysiology	119	64.5
0111117	Pathophysiology	119	62
0111117	Pathophysiology	119	82
0111117	Pathophysiology	119	84.5
0111117	Pathophysiology	119	78.25
0111117	Pathophysiology	119	59.5
0111117	Pathophysiology	119	54.5
0111117	Pathophysiology	119	55.75
0111117	Pathophysiology	119	53.25
0111117	Pathophysiology	119	78.25
0111117	Pathophysiology	119	59.5
0111117	Pathophysiology	119	52
0111117	Pathophysiology	119	57

0111117	Pathophysiology	119	60.75
0111117	Pathophysiology	119	75.75
0111117	Pathophysiology	119	72
0111117	Pathophysiology	119	57
0111117	Pathophysiology	119	58.25
0111117	Pathophysiology	119	80.75
0111117	Pathophysiology	119	54.5
0111117	Pathophysiology	119	69.5
0111117	Pathophysiology	119	54.5
0111117	Pathophysiology	119	65.75
0111117	Pathophysiology	119	60.75
0111117	Pathophysiology	119	90.75
0111117	Pathophysiology	119	60.75
0111117	Pathophysiology	119	68.25
0111117	Pathophysiology	119	58.25
0111117	Pathophysiology	119	80.75
0111117	Pathophysiology	119	78.25
0111117	Pathophysiology	119	62
mean score			66.79395604

120-122 Mid-term performance			
curriculum code	course	class	report card
0111117	Pathophysiology	120	63.25
0111117	Pathophysiology	120	69.5
0111117	Pathophysiology	120	75.75
0111117	Pathophysiology	120	39.5
0111117	Pathophysiology	120	72
0111117	Pathophysiology	120	87
0111117	Pathophysiology	120	74.5
0111117	Pathophysiology	120	44.5
0111117	Pathophysiology	120	78.25
0111117	Pathophysiology	120	79.5
0111117	Pathophysiology	120	83.25
0111117	Pathophysiology	120	55.75
0111117	Pathophysiology	120	40.75
0111117	Pathophysiology	120	39.5
0111117	Pathophysiology	120	65.75
0111117	Pathophysiology	120	59.5
0111117	Pathophysiology	120	75.75
0111117	Pathophysiology	120	63.25
0111117	Pathophysiology	120	72
0111117	Pathophysiology	120	78.25

0111117	Pathophysiology	120	73.25
0111117	Pathophysiology	120	65.75
0111117	Pathophysiology	120	63.25
0111117	Pathophysiology	120	59.5
0111117	Pathophysiology	120	62
0111117	Pathophysiology	120	84.5
0111117	Pathophysiology	120	65.75
0111117	Pathophysiology	120	73.25
0111117	Pathophysiology	120	65.75
0111117	Pathophysiology	120	82
0111117	Pathophysiology	120	63.25
0111117	Pathophysiology	121	83.25
0111117	Pathophysiology	121	69.5
0111117	Pathophysiology	121	50.5
0111117	Pathophysiology	121	63.25
0111117	Pathophysiology	121	70.75
0111117	Pathophysiology	121	68.25
0111117	Pathophysiology	121	63.25
0111117	Pathophysiology	121	75.75
0111117	Pathophysiology	121	48.25
0111117	Pathophysiology	121	74.5
0111117	Pathophysiology	121	80.75
0111117	Pathophysiology	121	82
0111117	Pathophysiology	121	83.25
0111117	Pathophysiology	121	63.25
0111117	Pathophysiology	121	47
0111117	Pathophysiology	121	82
0111117	Pathophysiology	121	85.75
0111117	Pathophysiology	121	83.25
0111117	Pathophysiology	121	63.25
0111117	Pathophysiology	121	60.5
0111117	Pathophysiology	121	82
0111117	Pathophysiology	121	59.5
0111117	Pathophysiology	121	70.75
0111117	Pathophysiology	121	33.25
0111117	Pathophysiology	121	77
0111117	Pathophysiology	121	83.25
0111117	Pathophysiology	121	83.25
0111117	Pathophysiology	121	78.25
0111117	Pathophysiology	121	34.5
0111117	Pathophysiology	121	59.5
0111117	Pathophysiology	122	55.75
0111117	Pathophysiology	122	79.5

0111117	Pathophysiology	122	75.75
0111117	Pathophysiology	122	37
0111117	Pathophysiology	122	82
0111117	Pathophysiology	122	50.75
0111117	Pathophysiology	122	70.75
0111117	Pathophysiology	122	58.25
0111117	Pathophysiology	122	59.5
0111117	Pathophysiology	122	57
0111117	Pathophysiology	122	72
0111117	Pathophysiology	122	59.5
0111117	Pathophysiology	122	62
0111117	Pathophysiology	122	67
0111117	Pathophysiology	122	72
0111117	Pathophysiology	122	68.25
0111117	Pathophysiology	122	52
0111117	Pathophysiology	122	62
0111117	Pathophysiology	122	65.75
0111117	Pathophysiology	122	65.75
0111117	Pathophysiology	122	40.75
0111117	Pathophysiology	122	52
0111117	Pathophysiology	122	50.75
0111117	Pathophysiology	122	63.25
0111117	Pathophysiology	122	72
0111117	Pathophysiology	122	65.75
0111117	Pathophysiology	122	87
0111117	Pathophysiology	122	82
0111117	Pathophysiology	122	60.5
0111117	Pathophysiology	122	69.5
mean score			66.49725275

17-19 Mid-term performance			
curriculum code	course	class	report card
0111117	Pathophysiology	17	75.75
0111117	Pathophysiology	17	79.5
0111117	Pathophysiology	17	54.5
0111117	Pathophysiology	17	67
0111117	Pathophysiology	17	72
0111117	Pathophysiology	17	75.75
0111117	Pathophysiology	17	42
0111117	Pathophysiology	17	67
0111117	Pathophysiology	17	72
0111117	Pathophysiology	17	72

0111117	Pathophysiology	17	72
0111117	Pathophysiology	17	55.75
0111117	Pathophysiology	17	68.25
0111117	Pathophysiology	17	88.25
0111117	Pathophysiology	17	89.5
0111117	Pathophysiology	17	73.25
0111117	Pathophysiology	17	63.25
0111117	Pathophysiology	17	74.5
0111117	Pathophysiology	17	72
0111117	Pathophysiology	17	54.5
0111117	Pathophysiology	17	83.25
0111117	Pathophysiology	17	70.75
0111117	Pathophysiology	17	82
0111117	Pathophysiology	17	95.75
0111117	Pathophysiology	17	82
0111117	Pathophysiology	17	50.75
0111117	Pathophysiology	17	50.75
0111117	Pathophysiology	17	55.75
0111117	Pathophysiology	17	65.75
0111117	Pathophysiology	17	65.75
0111117	Pathophysiology	17	33.25
0111117	Pathophysiology	17	55.75
0111117	Pathophysiology	17	50.75
0111117	Pathophysiology	17	83.25
0111117	Pathophysiology	17	68.25
0111117	Pathophysiology	18	74.5
0111117	Pathophysiology	18	67
0111117	Pathophysiology	18	74.5
0111117	Pathophysiology	18	85.75
0111117	Pathophysiology	18	67
0111117	Pathophysiology	18	87
0111117	Pathophysiology	18	88.25
0111117	Pathophysiology	18	0
0111117	Pathophysiology	18	74.5
0111117	Pathophysiology	18	92
0111117	Pathophysiology	18	92
0111117	Pathophysiology	18	63.25
0111117	Pathophysiology	18	86
0111117	Pathophysiology	18	79.5
0111117	Pathophysiology	18	87
0111117	Pathophysiology	18	77
0111117	Pathophysiology	18	64.5
0111117	Pathophysiology	18	67

0111117	Pathophysiology	18	74.5
0111117	Pathophysiology	18	80.75
0111117	Pathophysiology	18	74.5
0111117	Pathophysiology	18	78.25
0111117	Pathophysiology	18	42
0111117	Pathophysiology	18	80.75
0111117	Pathophysiology	18	55.75
0111117	Pathophysiology	18	78.25
0111117	Pathophysiology	18	93.25
0111117	Pathophysiology	18	82
0111117	Pathophysiology	18	83.25
0111117	Pathophysiology	18	69.5
0111117	Pathophysiology	18	58.25
0111117	Pathophysiology	18	88.25
0111117	Pathophysiology	18	54.5
0111117	Pathophysiology	18	62
0111117	Pathophysiology	18	79.5
0111117	Pathophysiology	19	49.5
0111117	Pathophysiology	19	67
0111117	Pathophysiology	19	64.5
0111117	Pathophysiology	19	62
0111117	Pathophysiology	19	47
0111117	Pathophysiology	19	65.75
0111117	Pathophysiology	19	59.75
0111117	Pathophysiology	19	79.5
0111117	Pathophysiology	19	88.25
0111117	Pathophysiology	19	65.75
0111117	Pathophysiology	19	78.25
0111117	Pathophysiology	19	58.25
0111117	Pathophysiology	19	77
0111117	Pathophysiology	19	63.25
0111117	Pathophysiology	19	70.75
0111117	Pathophysiology	19	68.25
0111117	Pathophysiology	19	47
0111117	Pathophysiology	19	65.75
0111117	Pathophysiology	19	79.5
0111117	Pathophysiology	19	47
0111117	Pathophysiology	19	50.75
0111117	Pathophysiology	19	74.5
0111117	Pathophysiology	19	39.5
0111117	Pathophysiology	19	44.5
0111117	Pathophysiology	19	62
0111117	Pathophysiology	19	49.5

0111117	Pathophysiology	19	93.25
0111117	Pathophysiology	19	52
0111117	Pathophysiology	19	44.5
0111117	Pathophysiology	19	54.5
0111117	Pathophysiology	19	63.25
0111117	Pathophysiology	19	55.75
0111117	Pathophysiology	19	43.25
0111117	Pathophysiology	19	50.75
mean score			67.5625

20-22 Mid-term performance			
curriculum code	course	class	report card
0111117	Pathophysiology	20	70.75
0111117	Pathophysiology	20	55.75
0111117	Pathophysiology	20	62
0111117	Pathophysiology	20	78.25
0111117	Pathophysiology	20	90.75
0111117	Pathophysiology	20	54.5
0111117	Pathophysiology	20	79.5
0111117	Pathophysiology	20	52
0111117	Pathophysiology	20	48.25
0111117	Pathophysiology	20	63.25
0111117	Pathophysiology	20	80.75
0111117	Pathophysiology	20	65.75
0111117	Pathophysiology	20	73.25
0111117	Pathophysiology	20	89.5
0111117	Pathophysiology	20	69.5
0111117	Pathophysiology	20	64.5
0111117	Pathophysiology	20	43.25
0111117	Pathophysiology	20	55.75
0111117	Pathophysiology	20	40.75
0111117	Pathophysiology	20	34.5
0111117	Pathophysiology	20	39.5
0111117	Pathophysiology	20	44.5
0111117	Pathophysiology	20	44.5
0111117	Pathophysiology	20	48.25
0111117	Pathophysiology	20	64.5
0111117	Pathophysiology	20	45.75
0111117	Pathophysiology	20	69.5

0111117	Pathophysiology	20	50.75
0111117	Pathophysiology	20	60.5
0111117	Pathophysiology	20	62
0111117	Pathophysiology	20	67
0111117	Pathophysiology	21	47
0111117	Pathophysiology	21	79.5
0111117	Pathophysiology	21	62
0111117	Pathophysiology	21	75.75
0111117	Pathophysiology	21	58.25
0111117	Pathophysiology	21	44.5
0111117	Pathophysiology	21	80.75
0111117	Pathophysiology	21	75.75
0111117	Pathophysiology	21	88.25
0111117	Pathophysiology	21	62
0111117	Pathophysiology	21	85.75
0111117	Pathophysiology	21	72
0111117	Pathophysiology	21	60.25
0111117	Pathophysiology	21	47
0111117	Pathophysiology	21	57
0111117	Pathophysiology	21	44.5
0111117	Pathophysiology	21	35.75
0111117	Pathophysiology	21	55.75
0111117	Pathophysiology	21	63.25
0111117	Pathophysiology	21	80.75
0111117	Pathophysiology	21	44.5
0111117	Pathophysiology	21	45.75
0111117	Pathophysiology	21	67.75
0111117	Pathophysiology	21	80.75
0111117	Pathophysiology	21	64.5
0111117	Pathophysiology	21	47
0111117	Pathophysiology	21	70.75
0111117	Pathophysiology	21	65.75
0111117	Pathophysiology	21	60.75
0111117	Pathophysiology	21	34.5
0111117	Pathophysiology	21	50.75
0111117	Pathophysiology	21	42
0111117	Pathophysiology	21	69.5
0111117	Pathophysiology	22	55.75
0111117	Pathophysiology	22	67
0111117	Pathophysiology	22	74.5
0111117	Pathophysiology	22	82
0111117	Pathophysiology	22	64.5
0111117	Pathophysiology	22	78.25

0111117	Pathophysiology	22	80.75
0111117	Pathophysiology	22	70.25
0111117	Pathophysiology	22	67
0111117	Pathophysiology	22	82
0111117	Pathophysiology	22	79.5
0111117	Pathophysiology	22	65.75
0111117	Pathophysiology	22	59.5
0111117	Pathophysiology	22	42
0111117	Pathophysiology	22	58.25
0111117	Pathophysiology	22	47
0111117	Pathophysiology	22	43.25
0111117	Pathophysiology	22	59.5
0111117	Pathophysiology	22	67
0111117	Pathophysiology	22	58.25
0111117	Pathophysiology	22	72
0111117	Pathophysiology	22	47
0111117	Pathophysiology	22	73.25
0111117	Pathophysiology	22	53.25
0111117	Pathophysiology	22	54.5
0111117	Pathophysiology	22	80.75
0111117	Pathophysiology	22	72
0111117	Pathophysiology	22	69.5
0111117	Pathophysiology	22	68.25
0111117	Pathophysiology	22	73.25
0111117	Pathophysiology	22	70.5
0111117	Pathophysiology	22	72
mean score			62.47395833

1-4 Final performance			
curriculum code	course	class	report card
0111117	Pathophysiology	1	89
0111117	Pathophysiology	1	76
0111117	Pathophysiology	1	93

0111117	Pathophysiology	1	83
0111117	Pathophysiology	1	68
0111117	Pathophysiology	1	77
0111117	Pathophysiology	1	93
0111117	Pathophysiology	1	67
0111117	Pathophysiology	1	77
0111117	Pathophysiology	1	90
0111117	Pathophysiology	1	86
0111117	Pathophysiology	1	87
0111117	Pathophysiology	1	69
0111117	Pathophysiology	1	68
0111117	Pathophysiology	1	68
0111117	Pathophysiology	1	76
0111117	Pathophysiology	1	94
0111117	Pathophysiology	1	65
0111117	Pathophysiology	1	67
0111117	Pathophysiology	1	70
0111117	Pathophysiology	1	68
0111117	Pathophysiology	1	74
0111117	Pathophysiology	1	75
0111117	Pathophysiology	1	69
0111117	Pathophysiology	1	64
0111117	Pathophysiology	1	72
0111117	Pathophysiology	1	56
0111117	Pathophysiology	1	82
0111117	Pathophysiology	1	69
0111117	Pathophysiology	1	70
0111117	Pathophysiology	2	87
0111117	Pathophysiology	2	95
0111117	Pathophysiology	2	78
0111117	Pathophysiology	2	77
0111117	Pathophysiology	2	88
0111117	Pathophysiology	2	77
0111117	Pathophysiology	2	76
0111117	Pathophysiology	2	78
0111117	Pathophysiology	2	69
0111117	Pathophysiology	2	83
0111117	Pathophysiology	2	66
0111117	Pathophysiology	2	57
0111117	Pathophysiology	2	69
0111117	Pathophysiology	2	78
0111117	Pathophysiology	2	74
0111117	Pathophysiology	2	76

0111117	Pathophysiology	2	75
0111117	Pathophysiology	2	79
0111117	Pathophysiology	2	65
0111117	Pathophysiology	2	73
0111117	Pathophysiology	2	76
0111117	Pathophysiology	2	75
0111117	Pathophysiology	2	64
0111117	Pathophysiology	2	85
0111117	Pathophysiology	2	75
0111117	Pathophysiology	2	72
0111117	Pathophysiology	2	79
0111117	Pathophysiology	2	74
0111117	Pathophysiology	2	79
0111117	Pathophysiology	2	70
0111117	Pathophysiology	3	70
0111117	Pathophysiology	3	75
0111117	Pathophysiology	3	74
0111117	Pathophysiology	3	84
0111117	Pathophysiology	3	86
0111117	Pathophysiology	3	88
0111117	Pathophysiology	3	65
0111117	Pathophysiology	3	80
0111117	Pathophysiology	3	67
0111117	Pathophysiology	3	77
0111117	Pathophysiology	3	89
0111117	Pathophysiology	3	75
0111117	Pathophysiology	3	73
0111117	Pathophysiology	3	67
0111117	Pathophysiology	3	79
0111117	Pathophysiology	3	69
0111117	Pathophysiology	3	78
0111117	Pathophysiology	3	89
0111117	Pathophysiology	3	68
0111117	Pathophysiology	3	75
0111117	Pathophysiology	3	80
0111117	Pathophysiology	3	89
0111117	Pathophysiology	3	69
0111117	Pathophysiology	3	76
0111117	Pathophysiology	3	82
0111117	Pathophysiology	3	82
0111117	Pathophysiology	3	68
0111117	Pathophysiology	3	69
0111117	Pathophysiology	3	65

0111117	Pathophysiology	3	62
0111117	Pathophysiology	4	84
0111117	Pathophysiology	4	80
0111117	Pathophysiology	4	69
0111117	Pathophysiology	4	74
0111117	Pathophysiology	4	80
0111117	Pathophysiology	4	77
0111117	Pathophysiology	4	78
0111117	Pathophysiology	4	91
0111117	Pathophysiology	4	85
0111117	Pathophysiology	4	87
0111117	Pathophysiology	4	65
0111117	Pathophysiology	4	84
0111117	Pathophysiology	4	83
0111117	Pathophysiology	4	78
0111117	Pathophysiology	4	95
0111117	Pathophysiology	4	78
0111117	Pathophysiology	4	65
0111117	Pathophysiology	4	76
0111117	Pathophysiology	4	89
0111117	Pathophysiology	4	79
0111117	Pathophysiology	4	75
0111117	Pathophysiology	4	78
0111117	Pathophysiology	4	89
0111117	Pathophysiology	4	85
0111117	Pathophysiology	4	66
0111117	Pathophysiology	4	85
0111117	Pathophysiology	4	67
0111117	Pathophysiology	4	83
0111117	Pathophysiology	4	79
0111117	Pathophysiology	4	78
0111117	Pathophysiology	4	83
0111117	Pathophysiology	4	67
mean score			76.5

5-8 Final performance			
curriculum code	course	class	report card
0111117	Pathophysiology	5	89
0111117	Pathophysiology	5	92
0111117	Pathophysiology	5	85

0111117	Pathophysiology	5	63
0111117	Pathophysiology	5	69
0111117	Pathophysiology	5	92
0111117	Pathophysiology	5	76
0111117	Pathophysiology	5	68
0111117	Pathophysiology	5	67
0111117	Pathophysiology	5	73
0111117	Pathophysiology	5	83
0111117	Pathophysiology	5	84
0111117	Pathophysiology	5	74
0111117	Pathophysiology	5	74
0111117	Pathophysiology	5	86
0111117	Pathophysiology	5	72
0111117	Pathophysiology	5	65
0111117	Pathophysiology	5	80
0111117	Pathophysiology	5	61
0111117	Pathophysiology	5	76
0111117	Pathophysiology	5	84
0111117	Pathophysiology	5	60
0111117	Pathophysiology	5	42
0111117	Pathophysiology	5	76
0111117	Pathophysiology	5	62
0111117	Pathophysiology	5	64
0111117	Pathophysiology	5	77
0111117	Pathophysiology	5	65
0111117	Pathophysiology	5	67
0111117	Pathophysiology	5	84
0111117	Pathophysiology	5	63
0111117	Pathophysiology	6	90
0111117	Pathophysiology	6	90
0111117	Pathophysiology	6	87
0111117	Pathophysiology	6	79
0111117	Pathophysiology	6	65
0111117	Pathophysiology	6	83
0111117	Pathophysiology	6	68
0111117	Pathophysiology	6	75
0111117	Pathophysiology	6	71
0111117	Pathophysiology	6	58
0111117	Pathophysiology	6	71
0111117	Pathophysiology	6	74
0111117	Pathophysiology	6	56
0111117	Pathophysiology	6	80
0111117	Pathophysiology	6	72

0111117	Pathophysiology	6	69
0111117	Pathophysiology	6	67
0111117	Pathophysiology	6	74
0111117	Pathophysiology	6	69
0111117	Pathophysiology	6	80
0111117	Pathophysiology	6	72
0111117	Pathophysiology	6	77
0111117	Pathophysiology	6	65
0111117	Pathophysiology	6	68
0111117	Pathophysiology	6	52
0111117	Pathophysiology	6	78
0111117	Pathophysiology	6	70
0111117	Pathophysiology	6	65
0111117	Pathophysiology	6	75
0111117	Pathophysiology	7	79
0111117	Pathophysiology	7	80
0111117	Pathophysiology	7	90
0111117	Pathophysiology	7	77
0111117	Pathophysiology	7	65
0111117	Pathophysiology	7	83
0111117	Pathophysiology	7	84
0111117	Pathophysiology	7	71
0111117	Pathophysiology	7	67
0111117	Pathophysiology	7	71
0111117	Pathophysiology	7	69
0111117	Pathophysiology	7	70
0111117	Pathophysiology	7	75
0111117	Pathophysiology	7	63
0111117	Pathophysiology	7	75
0111117	Pathophysiology	7	80
0111117	Pathophysiology	7	80
0111117	Pathophysiology	7	85
0111117	Pathophysiology	7	82
0111117	Pathophysiology	7	73
0111117	Pathophysiology	7	73
0111117	Pathophysiology	7	86
0111117	Pathophysiology	7	84
0111117	Pathophysiology	7	70
0111117	Pathophysiology	7	76
0111117	Pathophysiology	7	86
0111117	Pathophysiology	7	72
0111117	Pathophysiology	7	73
0111117	Pathophysiology	7	87

0111117	Pathophysiology	7	76
0111117	Pathophysiology	8	83
0111117	Pathophysiology	8	86
0111117	Pathophysiology	8	85
0111117	Pathophysiology	8	94
0111117	Pathophysiology	8	94
0111117	Pathophysiology	8	91
0111117	Pathophysiology	8	87
0111117	Pathophysiology	8	65
0111117	Pathophysiology	8	71
0111117	Pathophysiology	8	77
0111117	Pathophysiology	8	72
0111117	Pathophysiology	8	78
0111117	Pathophysiology	8	84
0111117	Pathophysiology	8	88
0111117	Pathophysiology	8	89
0111117	Pathophysiology	8	80
0111117	Pathophysiology	8	89
0111117	Pathophysiology	8	75
0111117	Pathophysiology	8	70
0111117	Pathophysiology	8	73
0111117	Pathophysiology	8	61
0111117	Pathophysiology	8	61
0111117	Pathophysiology	8	67
0111117	Pathophysiology	8	60
0111117	Pathophysiology	8	57
0111117	Pathophysiology	8	71
0111117	Pathophysiology	8	84
0111117	Pathophysiology	8	73
0111117	Pathophysiology	8	73
mean score			74.89915966

9-12 Final performance			
curriculum code	course	class	report card
0111117	Pathophysiology	9	84
0111117	Pathophysiology	9	77
0111117	Pathophysiology	9	82
0111117	Pathophysiology	9	77
0111117	Pathophysiology	9	88
0111117	Pathophysiology	9	89

0111117	Pathophysiology	9	81
0111117	Pathophysiology	9	89
0111117	Pathophysiology	9	75
0111117	Pathophysiology	9	78
0111117	Pathophysiology	9	74
0111117	Pathophysiology	9	76
0111117	Pathophysiology	9	76
0111117	Pathophysiology	9	74
0111117	Pathophysiology	9	78
0111117	Pathophysiology	9	77
0111117	Pathophysiology	9	78
0111117	Pathophysiology	9	64
0111117	Pathophysiology	9	77
0111117	Pathophysiology	9	77
0111117	Pathophysiology	9	86
0111117	Pathophysiology	9	85
0111117	Pathophysiology	9	72
0111117	Pathophysiology	9	87
0111117	Pathophysiology	9	77
0111117	Pathophysiology	9	73
0111117	Pathophysiology	9	79
0111117	Pathophysiology	9	74
0111117	Pathophysiology	9	64
0111117	Pathophysiology	9	67
0111117	Pathophysiology	9	76
0111117	Pathophysiology	9	72
0111117	Pathophysiology	9	72
0111117	Pathophysiology	9	77
0111117	Pathophysiology	10	68
0111117	Pathophysiology	10	76
0111117	Pathophysiology	10	89
0111117	Pathophysiology	10	79
0111117	Pathophysiology	10	79
0111117	Pathophysiology	10	78
0111117	Pathophysiology	10	80
0111117	Pathophysiology	10	62
0111117	Pathophysiology	10	68
0111117	Pathophysiology	10	65
0111117	Pathophysiology	10	76
0111117	Pathophysiology	10	80
0111117	Pathophysiology	10	78
0111117	Pathophysiology	10	74
0111117	Pathophysiology	10	35

0111117	Pathophysiology	10	81
0111117	Pathophysiology	10	76
0111117	Pathophysiology	10	84
0111117	Pathophysiology	10	75
0111117	Pathophysiology	10	55
0111117	Pathophysiology	10	60
0111117	Pathophysiology	10	69
0111117	Pathophysiology	10	63
0111117	Pathophysiology	10	72
0111117	Pathophysiology	10	68
0111117	Pathophysiology	10	78
0111117	Pathophysiology	10	71
0111117	Pathophysiology	10	70
0111117	Pathophysiology	10	78
0111117	Pathophysiology	10	76
0111117	Pathophysiology	10	68
0111117	Pathophysiology	11	69
0111117	Pathophysiology	11	82
0111117	Pathophysiology	11	87
0111117	Pathophysiology	11	87
0111117	Pathophysiology	11	65
0111117	Pathophysiology	11	82
0111117	Pathophysiology	11	93
0111117	Pathophysiology	11	87
0111117	Pathophysiology	11	86
0111117	Pathophysiology	11	89
0111117	Pathophysiology	11	58
0111117	Pathophysiology	11	74
0111117	Pathophysiology	11	76
0111117	Pathophysiology	11	68
0111117	Pathophysiology	11	79
0111117	Pathophysiology	11	69
0111117	Pathophysiology	11	90
0111117	Pathophysiology	11	71
0111117	Pathophysiology	11	84
0111117	Pathophysiology	11	80
0111117	Pathophysiology	11	90
0111117	Pathophysiology	11	78
0111117	Pathophysiology	11	69
0111117	Pathophysiology	11	84
0111117	Pathophysiology	11	82
0111117	Pathophysiology	11	70
0111117	Pathophysiology	11	84

0111117	Pathophysiology	11	66
0111117	Pathophysiology	11	78
0111117	Pathophysiology	11	71
0111117	Pathophysiology	11	84
0111117	Pathophysiology	11	87
0111117	Pathophysiology	12	73
0111117	Pathophysiology	12	63
0111117	Pathophysiology	12	79
0111117	Pathophysiology	12	92
0111117	Pathophysiology	12	82
0111117	Pathophysiology	12	79
0111117	Pathophysiology	12	69
0111117	Pathophysiology	12	79
0111117	Pathophysiology	12	89
0111117	Pathophysiology	12	76
0111117	Pathophysiology	12	87
0111117	Pathophysiology	12	77
0111117	Pathophysiology	12	67
0111117	Pathophysiology	12	68
0111117	Pathophysiology	12	71
0111117	Pathophysiology	12	66
0111117	Pathophysiology	12	78
0111117	Pathophysiology	12	66
0111117	Pathophysiology	12	70
0111117	Pathophysiology	12	76
0111117	Pathophysiology	12	79
0111117	Pathophysiology	12	76
0111117	Pathophysiology	12	74
0111117	Pathophysiology	12	69
0111117	Pathophysiology	12	56
0111117	Pathophysiology	12	75
0111117	Pathophysiology	12	73
0111117	Pathophysiology	12	67
0111117	Pathophysiology	12	69
0111117	Pathophysiology	12	67
mean score			75.54330709

13-16 Final performance			
curriculum code	course	class	report card
0111117	Pathophysiology	13	61

0111117	Pathophysiology	13	67
0111117	Pathophysiology	13	82
0111117	Pathophysiology	13	72
0111117	Pathophysiology	13	83
0111117	Pathophysiology	13	85
0111117	Pathophysiology	13	86
0111117	Pathophysiology	13	81
0111117	Pathophysiology	13	80
0111117	Pathophysiology	13	72
0111117	Pathophysiology	13	96
0111117	Pathophysiology	13	91
0111117	Pathophysiology	13	77
0111117	Pathophysiology	13	86
0111117	Pathophysiology	13	81
0111117	Pathophysiology	13	66
0111117	Pathophysiology	13	67
0111117	Pathophysiology	13	75
0111117	Pathophysiology	13	70
0111117	Pathophysiology	13	75
0111117	Pathophysiology	13	85
0111117	Pathophysiology	13	77
0111117	Pathophysiology	13	86
0111117	Pathophysiology	13	66
0111117	Pathophysiology	13	75
0111117	Pathophysiology	13	87
0111117	Pathophysiology	13	70
0111117	Pathophysiology	13	65
0111117	Pathophysiology	13	78
0111117	Pathophysiology	13	83
0111117	Pathophysiology	13	62
0111117	Pathophysiology	14	93
0111117	Pathophysiology	14	89
0111117	Pathophysiology	14	79
0111117	Pathophysiology	14	60
0111117	Pathophysiology	14	73
0111117	Pathophysiology	14	89
0111117	Pathophysiology	14	70
0111117	Pathophysiology	14	82
0111117	Pathophysiology	14	82
0111117	Pathophysiology	14	80
0111117	Pathophysiology	14	87
0111117	Pathophysiology	14	67
0111117	Pathophysiology	14	85

0111117	Pathophysiology	14	86
0111117	Pathophysiology	14	80
0111117	Pathophysiology	14	68
0111117	Pathophysiology	14	74
0111117	Pathophysiology	14	67
0111117	Pathophysiology	14	85
0111117	Pathophysiology	14	73
0111117	Pathophysiology	14	73
0111117	Pathophysiology	14	74
0111117	Pathophysiology	14	78
0111117	Pathophysiology	14	80
0111117	Pathophysiology	14	64
0111117	Pathophysiology	14	61
0111117	Pathophysiology	14	70
0111117	Pathophysiology	14	68
0111117	Pathophysiology	14	66
0111117	Pathophysiology	14	68
0111117	Pathophysiology	14	77
0111117	Pathophysiology	15	80
0111117	Pathophysiology	15	83
0111117	Pathophysiology	15	68
0111117	Pathophysiology	15	73
0111117	Pathophysiology	15	60
0111117	Pathophysiology	15	75
0111117	Pathophysiology	15	77
0111117	Pathophysiology	15	74
0111117	Pathophysiology	15	81
0111117	Pathophysiology	15	64
0111117	Pathophysiology	15	77
0111117	Pathophysiology	15	92
0111117	Pathophysiology	15	73
0111117	Pathophysiology	15	78
0111117	Pathophysiology	15	64
0111117	Pathophysiology	15	86
0111117	Pathophysiology	15	68
0111117	Pathophysiology	15	73
0111117	Pathophysiology	15	73
0111117	Pathophysiology	15	70
0111117	Pathophysiology	15	64
0111117	Pathophysiology	15	60
0111117	Pathophysiology	15	68
0111117	Pathophysiology	15	67
0111117	Pathophysiology	15	69

0111117	Pathophysiology	15	58
0111117	Pathophysiology	15	79
0111117	Pathophysiology	15	71
0111117	Pathophysiology	15	74
0111117	Pathophysiology	15	71
0111117	Pathophysiology	15	71
0111117	Pathophysiology	16	93
0111117	Pathophysiology	16	74
0111117	Pathophysiology	16	65
0111117	Pathophysiology	16	79
0111117	Pathophysiology	16	73
0111117	Pathophysiology	16	87
0111117	Pathophysiology	16	72
0111117	Pathophysiology	16	67
0111117	Pathophysiology	16	72
0111117	Pathophysiology	16	70
0111117	Pathophysiology	16	61
0111117	Pathophysiology	16	87
0111117	Pathophysiology	16	60
0111117	Pathophysiology	16	75
0111117	Pathophysiology	16	68
0111117	Pathophysiology	16	71
0111117	Pathophysiology	16	69
0111117	Pathophysiology	16	85
0111117	Pathophysiology	16	71
0111117	Pathophysiology	16	66
0111117	Pathophysiology	16	76
0111117	Pathophysiology	16	68
0111117	Pathophysiology	16	65
0111117	Pathophysiology	16	65
0111117	Pathophysiology	16	61
0111117	Pathophysiology	16	71
0111117	Pathophysiology	16	68
0111117	Pathophysiology	16	80
0111117	Pathophysiology	16	69
0111117	Pathophysiology	16	69
0111117	Pathophysiology	16	66
0111117	Pathophysiology	16	69
mean score			74.144

117-119 Final performance			
curriculum	course	class	report card

code			
0111117	Pathophysiology	117	90
0111117	Pathophysiology	117	76
0111117	Pathophysiology	117	65
0111117	Pathophysiology	117	79
0111117	Pathophysiology	117	72
0111117	Pathophysiology	117	84
0111117	Pathophysiology	117	81
0111117	Pathophysiology	117	90
0111117	Pathophysiology	117	64
0111117	Pathophysiology	117	65
0111117	Pathophysiology	117	75
0111117	Pathophysiology	117	89
0111117	Pathophysiology	117	79
0111117	Pathophysiology	117	68
0111117	Pathophysiology	117	87
0111117	Pathophysiology	117	73
0111117	Pathophysiology	117	60
0111117	Pathophysiology	117	82
0111117	Pathophysiology	117	70
0111117	Pathophysiology	117	89
0111117	Pathophysiology	117	73
0111117	Pathophysiology	117	69
0111117	Pathophysiology	117	85
0111117	Pathophysiology	117	81
0111117	Pathophysiology	117	85
0111117	Pathophysiology	117	72
0111117	Pathophysiology	117	75
0111117	Pathophysiology	117	76
0111117	Pathophysiology	117	73
0111117	Pathophysiology	117	86
0111117	Pathophysiology	118	89
0111117	Pathophysiology	118	70
0111117	Pathophysiology	118	78
0111117	Pathophysiology	118	78
0111117	Pathophysiology	118	79
0111117	Pathophysiology	118	71
0111117	Pathophysiology	118	74
0111117	Pathophysiology	118	89
0111117	Pathophysiology	118	75
0111117	Pathophysiology	118	77
0111117	Pathophysiology	118	74
0111117	Pathophysiology	118	82

0111117	Pathophysiology	118	78
0111117	Pathophysiology	118	85
0111117	Pathophysiology	118	68
0111117	Pathophysiology	118	84
0111117	Pathophysiology	118	72
0111117	Pathophysiology	118	66
0111117	Pathophysiology	118	74
0111117	Pathophysiology	118	82
0111117	Pathophysiology	118	56
0111117	Pathophysiology	118	78
0111117	Pathophysiology	118	81
0111117	Pathophysiology	118	89
0111117	Pathophysiology	118	66
0111117	Pathophysiology	118	83
0111117	Pathophysiology	118	77
0111117	Pathophysiology	118	74
0111117	Pathophysiology	118	90
0111117	Pathophysiology	118	74
0111117	Pathophysiology	119	68
0111117	Pathophysiology	119	75
0111117	Pathophysiology	119	74
0111117	Pathophysiology	119	83
0111117	Pathophysiology	119	78
0111117	Pathophysiology	119	68
0111117	Pathophysiology	119	89
0111117	Pathophysiology	119	90
0111117	Pathophysiology	119	79
0111117	Pathophysiology	119	81
0111117	Pathophysiology	119	89
0111117	Pathophysiology	119	66
0111117	Pathophysiology	119	79
0111117	Pathophysiology	119	75
0111117	Pathophysiology	119	74
0111117	Pathophysiology	119	70
0111117	Pathophysiology	119	76
0111117	Pathophysiology	119	80
0111117	Pathophysiology	119	89
0111117	Pathophysiology	119	74
0111117	Pathophysiology	119	73
0111117	Pathophysiology	119	78
0111117	Pathophysiology	119	84
0111117	Pathophysiology	119	75
0111117	Pathophysiology	119	79

0111117	Pathophysiology	119	75
0111117	Pathophysiology	119	77
0111117	Pathophysiology	119	74
0111117	Pathophysiology	119	89
0111117	Pathophysiology	119	79
0111117	Pathophysiology	119	90
mean score			77.53846154

120-122 Final performance			
curriculum code	course	class	report card
0111117	Pathophysiology	120	79
0111117	Pathophysiology	120	76
0111117	Pathophysiology	120	65
0111117	Pathophysiology	120	75
0111117	Pathophysiology	120	72
0111117	Pathophysiology	120	84
0111117	Pathophysiology	120	81
0111117	Pathophysiology	120	90
0111117	Pathophysiology	120	64
0111117	Pathophysiology	120	65
0111117	Pathophysiology	120	75
0111117	Pathophysiology	120	81
0111117	Pathophysiology	120	79
0111117	Pathophysiology	120	68
0111117	Pathophysiology	120	87
0111117	Pathophysiology	120	73
0111117	Pathophysiology	120	60
0111117	Pathophysiology	120	82
0111117	Pathophysiology	120	70
0111117	Pathophysiology	120	89
0111117	Pathophysiology	120	73
0111117	Pathophysiology	120	69
0111117	Pathophysiology	120	85
0111117	Pathophysiology	120	81
0111117	Pathophysiology	120	85
0111117	Pathophysiology	120	72
0111117	Pathophysiology	120	75
0111117	Pathophysiology	120	76
0111117	Pathophysiology	120	73
0111117	Pathophysiology	120	86
0111117	Pathophysiology	120	70

0111117	Pathophysiology	121	70
0111117	Pathophysiology	121	78
0111117	Pathophysiology	121	78
0111117	Pathophysiology	121	79
0111117	Pathophysiology	121	71
0111117	Pathophysiology	121	74
0111117	Pathophysiology	121	89
0111117	Pathophysiology	121	75
0111117	Pathophysiology	121	77
0111117	Pathophysiology	121	74
0111117	Pathophysiology	121	82
0111117	Pathophysiology	121	78
0111117	Pathophysiology	121	85
0111117	Pathophysiology	121	68
0111117	Pathophysiology	121	84
0111117	Pathophysiology	121	72
0111117	Pathophysiology	121	66
0111117	Pathophysiology	121	74
0111117	Pathophysiology	121	82
0111117	Pathophysiology	121	56
0111117	Pathophysiology	121	71
0111117	Pathophysiology	121	81
0111117	Pathophysiology	121	80
0111117	Pathophysiology	121	66
0111117	Pathophysiology	121	83
0111117	Pathophysiology	121	77
0111117	Pathophysiology	121	74
0111117	Pathophysiology	121	90
0111117	Pathophysiology	121	74
0111117	Pathophysiology	121	68
0111117	Pathophysiology	122	75
0111117	Pathophysiology	122	74
0111117	Pathophysiology	122	83
0111117	Pathophysiology	122	78
0111117	Pathophysiology	122	68
0111117	Pathophysiology	122	73
0111117	Pathophysiology	122	73
0111117	Pathophysiology	122	70
0111117	Pathophysiology	122	81
0111117	Pathophysiology	122	75
0111117	Pathophysiology	122	63
0111117	Pathophysiology	122	79
0111117	Pathophysiology	122	75

0111117	Pathophysiology	122	74
0111117	Pathophysiology	122	70
0111117	Pathophysiology	122	67
0111117	Pathophysiology	122	80
0111117	Pathophysiology	122	81
0111117	Pathophysiology	122	74
0111117	Pathophysiology	122	73
0111117	Pathophysiology	122	72
0111117	Pathophysiology	122	84
0111117	Pathophysiology	122	75
0111117	Pathophysiology	122	79
0111117	Pathophysiology	122	70
0111117	Pathophysiology	122	77
0111117	Pathophysiology	122	74
0111117	Pathophysiology	122	71
0111117	Pathophysiology	122	70
0111117	Pathophysiology	122	73
mean score			75.46153846

17-19 Final performance			
curriculum code	course	class	report card
0111117	Pathophysiology	17	76
0111117	Pathophysiology	17	86
0111117	Pathophysiology	17	68
0111117	Pathophysiology	17	85
0111117	Pathophysiology	17	80
0111117	Pathophysiology	17	84
0111117	Pathophysiology	17	67
0111117	Pathophysiology	17	81
0111117	Pathophysiology	17	84
0111117	Pathophysiology	17	82
0111117	Pathophysiology	17	77
0111117	Pathophysiology	17	69
0111117	Pathophysiology	17	79
0111117	Pathophysiology	17	94
0111117	Pathophysiology	17	81
0111117	Pathophysiology	17	80
0111117	Pathophysiology	17	73
0111117	Pathophysiology	17	84
0111117	Pathophysiology	17	73
0111117	Pathophysiology	17	69

0111117	Pathophysiology	17	89
0111117	Pathophysiology	17	66
0111117	Pathophysiology	17	82
0111117	Pathophysiology	17	90
0111117	Pathophysiology	17	84
0111117	Pathophysiology	17	71
0111117	Pathophysiology	17	70
0111117	Pathophysiology	17	50
0111117	Pathophysiology	17	69
0111117	Pathophysiology	17	76
0111117	Pathophysiology	17	62
0111117	Pathophysiology	17	63
0111117	Pathophysiology	17	67
0111117	Pathophysiology	17	82
0111117	Pathophysiology	17	75
0111117	Pathophysiology	18	79
0111117	Pathophysiology	18	78
0111117	Pathophysiology	18	70
0111117	Pathophysiology	18	89
0111117	Pathophysiology	18	75
0111117	Pathophysiology	18	87
0111117	Pathophysiology	18	84
0111117	Pathophysiology	18	60
0111117	Pathophysiology	18	85
0111117	Pathophysiology	18	85
0111117	Pathophysiology	18	93
0111117	Pathophysiology	18	70
0111117	Pathophysiology	18	83
0111117	Pathophysiology	18	89
0111117	Pathophysiology	18	66
0111117	Pathophysiology	18	79
0111117	Pathophysiology	18	72
0111117	Pathophysiology	18	70
0111117	Pathophysiology	18	72
0111117	Pathophysiology	18	86
0111117	Pathophysiology	18	76
0111117	Pathophysiology	18	86
0111117	Pathophysiology	18	70
0111117	Pathophysiology	18	82
0111117	Pathophysiology	18	67
0111117	Pathophysiology	18	82
0111117	Pathophysiology	18	82
0111117	Pathophysiology	18	87

0111117	Pathophysiology	18	82
0111117	Pathophysiology	18	78
0111117	Pathophysiology	18	68
0111117	Pathophysiology	18	79
0111117	Pathophysiology	18	75
0111117	Pathophysiology	18	76
0111117	Pathophysiology	18	84
0111117	Pathophysiology	19	70
0111117	Pathophysiology	19	80
0111117	Pathophysiology	19	76
0111117	Pathophysiology	19	73
0111117	Pathophysiology	19	73
0111117	Pathophysiology	19	76
0111117	Pathophysiology	19	76
0111117	Pathophysiology	19	82
0111117	Pathophysiology	19	87
0111117	Pathophysiology	19	78
0111117	Pathophysiology	19	81
0111117	Pathophysiology	19	74
0111117	Pathophysiology	19	81
0111117	Pathophysiology	19	78
0111117	Pathophysiology	19	77
0111117	Pathophysiology	19	74
0111117	Pathophysiology	19	60
0111117	Pathophysiology	19	73
0111117	Pathophysiology	19	79
0111117	Pathophysiology	19	66
0111117	Pathophysiology	19	56
0111117	Pathophysiology	19	78
0111117	Pathophysiology	19	67
0111117	Pathophysiology	19	65
0111117	Pathophysiology	19	65
0111117	Pathophysiology	19	56
0111117	Pathophysiology	19	83
0111117	Pathophysiology	19	73
0111117	Pathophysiology	19	60
0111117	Pathophysiology	19	69
0111117	Pathophysiology	19	78
0111117	Pathophysiology	19	73
0111117	Pathophysiology	19	67
0111117	Pathophysiology	19	62
mean			75.76923077

score			

20-22 Final performance			
curriculum code	course	class	report card
0111117	Pathophysiology	20	81
0111117	Pathophysiology	20	73
0111117	Pathophysiology	20	70
0111117	Pathophysiology	20	85
0111117	Pathophysiology	20	87
0111117	Pathophysiology	20	62
0111117	Pathophysiology	20	83
0111117	Pathophysiology	20	73
0111117	Pathophysiology	20	65
0111117	Pathophysiology	20	83
0111117	Pathophysiology	20	91
0111117	Pathophysiology	20	77
0111117	Pathophysiology	20	70
0111117	Pathophysiology	20	78
0111117	Pathophysiology	20	81
0111117	Pathophysiology	20	79
0111117	Pathophysiology	20	68
0111117	Pathophysiology	20	70
0111117	Pathophysiology	20	64
0111117	Pathophysiology	20	64
0111117	Pathophysiology	20	65
0111117	Pathophysiology	20	60
0111117	Pathophysiology	20	55
0111117	Pathophysiology	20	54
0111117	Pathophysiology	20	75
0111117	Pathophysiology	20	62
0111117	Pathophysiology	20	78
0111117	Pathophysiology	20	64
0111117	Pathophysiology	20	70
0111117	Pathophysiology	20	61
0111117	Pathophysiology	20	76
0111117	Pathophysiology	21	75
0111117	Pathophysiology	21	87
0111117	Pathophysiology	21	73
0111117	Pathophysiology	21	76
0111117	Pathophysiology	21	71

0111117	Pathophysiology	21	64
0111117	Pathophysiology	21	86
0111117	Pathophysiology	21	78
0111117	Pathophysiology	21	80
0111117	Pathophysiology	21	75
0111117	Pathophysiology	21	78
0111117	Pathophysiology	21	81
0111117	Pathophysiology	21	73
0111117	Pathophysiology	21	73
0111117	Pathophysiology	21	74
0111117	Pathophysiology	21	62
0111117	Pathophysiology	21	58
0111117	Pathophysiology	21	72
0111117	Pathophysiology	21	76
0111117	Pathophysiology	21	87
0111117	Pathophysiology	21	58
0111117	Pathophysiology	21	73
0111117	Pathophysiology	21	80
0111117	Pathophysiology	21	87
0111117	Pathophysiology	21	74
0111117	Pathophysiology	21	73
0111117	Pathophysiology	21	61
0111117	Pathophysiology	21	75
0111117	Pathophysiology	21	75
0111117	Pathophysiology	21	65
0111117	Pathophysiology	21	64
0111117	Pathophysiology	21	64
0111117	Pathophysiology	21	78
0111117	Pathophysiology	22	81
0111117	Pathophysiology	22	86
0111117	Pathophysiology	22	85
0111117	Pathophysiology	22	86
0111117	Pathophysiology	22	71
0111117	Pathophysiology	22	82
0111117	Pathophysiology	22	80
0111117	Pathophysiology	22	71
0111117	Pathophysiology	22	70
0111117	Pathophysiology	22	83
0111117	Pathophysiology	22	80
0111117	Pathophysiology	22	72
0111117	Pathophysiology	22	70
0111117	Pathophysiology	22	72
0111117	Pathophysiology	22	71

0111117	Pathophysiology	22	72
0111117	Pathophysiology	22	60
0111117	Pathophysiology	22	72
0111117	Pathophysiology	22	74
0111117	Pathophysiology	22	61
0111117	Pathophysiology	22	74
0111117	Pathophysiology	22	75
0111117	Pathophysiology	22	65
0111117	Pathophysiology	22	70
0111117	Pathophysiology	22	78
0111117	Pathophysiology	22	80
0111117	Pathophysiology	22	82
0111117	Pathophysiology	22	73
0111117	Pathophysiology	22	80
0111117	Pathophysiology	22	71
0111117	Pathophysiology	22	73
0111117	Pathophysiology	22	82
mean score			73.45833333
