# Supplementary material for: A comparative analysis of academic outcomes in blended versus traditional instructional approaches: An examination within the context of the National Medical Licensing Examination
Source: PLoS One. 2026 Apr 17;21(4):e0346793. doi: 10.1371/journal.pone.0346793 (PMC13089738; doi:10.1371/journal.pone.0346793)
Supplement: S5 File — (PDF) [file pone.0346793.s006.pdf]

Explain：The results of the questionnaire survey were obtained through a direct reliability analysis conducted on the website (with screenshots of the website attached), and the original questionnaire is shown below.

Sample capacity	number	Cronbach's aerfa
342	6	0.725

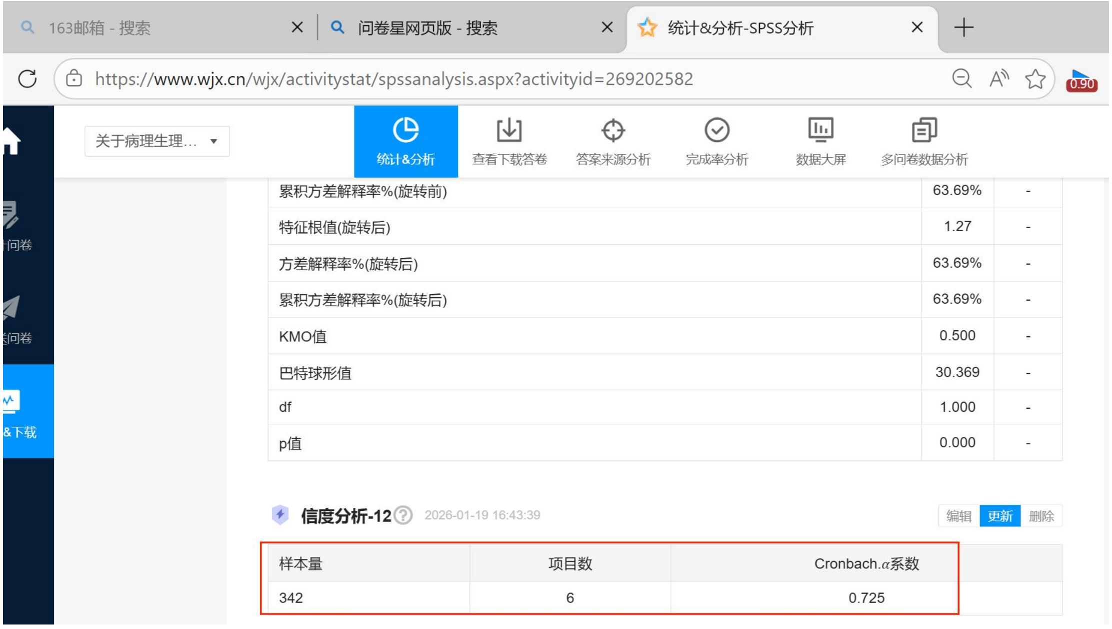

ENGLISH QUESTIONNAIRE

1.So far, have you adapted to the classroom model of blended teaching in Pathophysiology?

Choices	amount	Percentage(%)
A. Completely adapted	172	43.88
B. Adapted	138	35.2
C. Somewhat unadapted	28	7.14
D. Unadapted	4	1.02
E. Black	50	12.76

2.Which teaching resources do you prefer to use in the study of Pathophysiology?

Choices	amount	Percentage(%)
A. Textbook	351	89.54
B. Videos on Bilibili and other platforms	278	70.92
C. Resources pushed by Xuetangyun	207	52.81
D. Learning resources of Youmu courses	164	41.84
E. Others (please specify)	10	2.55

3.After completing the learning resources pushed by Xuetangyun (online learning platform), can you independently finish the practice questions in the courseware?

Choices	amount	Percentage(%)
A. Able to	301	76.79
B. Unable to	25	6.38
C. Unsure	66	16.84

4.Do you think you have a clear understanding of the content related to Pathophysiology in the National Medical Licensing Examination syllabus so far?

Choices	amount	Percentage(%)
A. Very clear	153	39.03
B. Clear	113	28.83
C. Roughly clear	100	25,51
D. Unclear	26	6.63

5.Do you think the study of Pathophysiology helps you understand relevant clinical knowledge?

Choices	amount	Percentage(%)
A. Very helpful	256	65.31
B. Helpful	131	33.42
C. Not very helpful	5	1.28

D. Not helpful	0	0

6.What capabilities do you think blended teaching has improved for you?

Choices	amount	Percentage(%)
A. Self - learning ability	274	69.9
B.Communication and expression ability	23	5.87
C. Independent thinking ability	87	22.19
D. Other abilities [Please specify]	2	0.51
E. No improvement	6	1.53

9.Are you satisfied with the teaching of Pathophysiology?

Choices	amount	Percentage(%)
A. Very satisfied	267	68.11
B. Satisfied	100	25.51
C. Basically satisfied	24	6.12
D. Dissatisfied	1	0,26
