# Supplementary material for: A comparative analysis of academic outcomes in blended versus traditional instructional approaches: An examination within the context of the National Medical Licensing Examination
Source: PLoS One. 2026 Apr 17;21(4):e0346793. doi: 10.1371/journal.pone.0346793 (PMC13089738; doi:10.1371/journal.pone.0346793)
Supplement: Attachment — Submitted filename: Response to Reviewers 1.7.docx [file pone.0346793.s009.docx]

Response Letter

Manuscript Number:PONE-D-25-34517.

Title:***A comparative analysis of academic outcomes in blended versus traditional instructional approaches: An examination within the context of the National Medical Licensing Examination.***

Nov. 21th, 2025

Dear Editor Dr.Ghilan Al Madhagy Ghilan Taufiq Hail, Reviewer Dr.Devisakti and Reviewers:

On behalf of my co-authors, we thank you for giving us a chance to revise and improve the quality of our article.

We have read the reviewers' and your comments carefully and have made revision which marked in red in the paper. We have tried our best to revise our manuscript according to the comments: “A Comparative Analysis of Academic Outcomes in Blended versus Traditional Instructional Approaches: An Examination within the Context of the National Medical Licensing Examination. (PONE-D-25-34517)”.

Attached please find the revised version, which we hope that you will find this updated manuscript to your satisfaction and consider it for publication as an article in *PLOS ONE*. Here is a point-by-point response to the reviewers' comments and concerns.

Thank you for taking the time to consider our research and we look forward to hearing from you at your earliest convenience.

Sincerely,

Siyu Zhang

North Henan Medical University,

Xinxiang, HeNan China

E－mail:shiyu15937135473@163.com

Comments from the Editor :

After careful consideration, we feel that it has merit but does not fully meet PLOS ONE’s publication criteria as it currently stands. Therefore, we invite you to submit a revised version of the manuscript that addresses the points raised during the review process.

1.Kindly adhere to the formatting of the journal

2.Check the language and revise the manuscript as indicated by one

of the reviewers

3.Revise the manuscript and comply with the reviewers comments in order to ensure that the manuscript is complying with the ethical standards as well as scientific research standards.

4.Please submit your revised manuscript by Dec 6,2025.

**Response:** We thank all the editors and reviewers for their valuable comments and suggestions. We have carefully revised the manuscript to enhance its clarity and facilitate the understanding of the readers. Our point-to-point responses are presented in the following. We hope that the revision would satisfactorily address the comments and concerns of the editors and reviewers.

Journal Requirements:

1. Please ensure that your manuscript meets PLOS ONE's style requirements, including those for file naming.

**Response:** Thank you foe your feedback.we have checked and revised the manuscript according to PLOS ONE's style requirements and confirmed that the file naming and format meets PLOS ONE's style.

2. Please provide additional details regarding participant consent. In the ethics statement in the Methods and online submission information, please ensure that you have specified (1) whether consent was informed and (2) what type you obtained (for instance, written or verbal, and if verbal, how it was documented and witnessed). If your study included minors, state whether you obtained consent from parents or guardians. If the need for consent was waived by the ethics committee, please include this information.

If you are reporting a retrospective study of medical records or archived samples, please ensure that you have discussed whether all data were fully anonymized before you accessed them and/or whether the IRB or ethics committee waived the requirement for informed consent. If patients provided informed written consent to have data from their medical records used in research, please include this information.

**Response:**We thank the reviewer for this important question regarding research ethics. This study involved the analysis of anonymized academic performance data and anonymized survey responses that were collected as part of a routine educational evaluation of the teaching methods. No personal identifying information was accessed or analyzed.We need to explain to you the issue regarding the use of student grades to obtain their informed consent. In China, after each exam, schools require teachers to conduct a grade analysis based on the students' scores. This grade analysis is then shared with the students, and Chinese students are informed.In the Chinese education system, analyzing students' grades to improve teaching quality is a very common practice. Chinese students are informed of this from the very beginning of their schooling.

3. We note that the grant information you provided in the ‘Funding Information’ and ‘Financial Disclosure’ sections do not match.
   When you resubmit, please ensure that you provide the correct grant numbers for the awards you received for your study in the ‘Funding Information’ section.

**Response:**Thank you for your reminder,we have revised it the latest manuscript.And we will explain the “Financial Disclosureissues” issues one by one in the fourth question.

4. Thank you for stating the following financial disclosure:
“This study was supported by 1. University-Level Teaching Reform Initiative at Yubei Medical College: An Empirical Investigation into the Blended Teaching Approach for Pathophysiology with a Focus on the Medical Licensing Examination (2023XJJG35). 2. "One Hospital, One Brand" Initiative by the Basic Medical College at Yubei Medical College: (yyyp2024001). 3. Henan Provincial Social Sciences Association: A Study on Developing High-Quality Faculty Teams in Medical Colleges Inspired by the Educators' Ethos (SKL-2024-805). 4. Outstanding Undergraduate Research Project Fund (2024002ZK, 2024004ZK, 2024008ZK, 2024031SK). 5. Fund for the Cultivation of Outstanding Young Teachers （SQ2022YQJH02）”

Please state what role the funders took in the study. If the funders had no role, please state: "The funders had no role in study design, data collection and analysis, decision to publish, or preparation of the manuscript."
If this statement is not correct you must amend it as needed.
Please include this amended Role of Funder statement in your cover letter; we will change the online submission form on your behalf.

**Response:**Thanks for your careful checks.We are sorry for our carelessness.Based on your comments,we had explained the following financial disclosure:

1. University-Level Teaching Reform Initiative at North Henan Medical University: An Empirical Investigation into the Blended Teaching Approach for Pathophysiology with a Focus on the Medical Licensing Examination (2023XJJG35) ; the funder is Siyu Zhang（the first author of the article had a role in preparation of the manuscript);

2. "One Hospital, One Brand" Initiative by the Basic Medical College at North Henan Medical University: (yyyp2024001); the funder is Baosheng Yang（The corresponding Author of the article had a role in study design);

3. Henan Provincial Social Sciences Association: A Study on Developing High-Quality Faculty Teams in Medical Colleges Inspired by the Educators' Ethos (SKL-2024-805) ; the funder is Peiji Wang（the second author of the article had a role in data collection );

4. Outstanding Undergraduate Research Project Fund (2024002ZK) ; the funder is Yuanjie Chen（the author of the article had a role in data analysis);Outstanding Undergraduate Research Project Fund( 2024004ZK, 2024008ZK, 2024031SK); the funder is Jinwen Sima（the author of the article had a role in data analysis);

5.Fund for the Cultivation of Outstanding Young Teachers （SQ2022YQJH02）; the funder is Peiji Wang（the second author of the article had a role in data collection ).

5. PLOS requires an ORCID iD for the corresponding author in Editorial Manager on papers submitted after December 6th, 2016. Please ensure that you have an ORCID iD and that it is validated in Editorial Manager. To do this, go to ‘Update my Information’ (in the upper left-hand corner of the main menu), and click on the Fetch/Validate link next to the ORCID field. This will take you to the ORCID site and allow you to create a new iD or authenticate a pre-existing iD in Editorial Manager.

**Response:**Thank you for your reminder.We had ensureed that we have an ORCID iD and that it is validated in Editorial Manager.

6. Please include captions for your Supporting Information files at the end of your manuscript, and update any in-text citations to match accordingly. Please see our Supporting Information guidelines for more information: <http://journals.plos.org/plosone/s/supporting-information.>

**Response:**We feel great thanks for your professional review work on our article. We had included captions for the Supporting Information files at the end of revised manuscript,and updated any in-text citations to match accordingly.The part on page 31 , lines 655-644 has been revised as follows:

Supporting information

S1 Fig.Instructional Design is Fig 1 Title. Schematic diagram of the blended teaching process integrating online and offline activities.The diagram illustrates the sequential teaching workflow across three phases: Before Class, In-Class, and After Class. Blue boxes represent teacher activities, while pink boxes represent corresponding student activities. The arrows indicate the progression of time and the interactive logic between teaching and learning steps. (PQE: Physician Qualification Examination) is the S1 Fig legend.

S1 File.Funding Statement is File1 Title.

S2 File.Questionnaire Survey Supplementary Material is File2 Title.

S3 File.Example of Pathophysiology Knowledge Graph is File3 Title.

S4 File.Research data is File4 Title.Midterm Scores, Final Scores, and Average Scores are the S4 File legend.

S5 File.Grading of Test Difficulty Coefficients is File5 Title.

S6 File.Original questionnaire is File5 Title.

7. If the reviewer comments include a recommendation to cite specific previously published works, please review and evaluate these publications to determine whether they are relevant and should be cited. There is no requirement to cite these works unless the editor has indicated otherwise.

**Response:**We appreciate your comment. We would review and evaluate these publications to determine whether the previously published works are relevant and should be cited. We will cite it with caution.

Reviewers' comments:

1. Is the manuscript technically sound, and do the data support the conclusions?

Reviewer #1: Yes

Reviewer #2: Yes

**Response:**We thank the two reviewer for the helpful comments and appreciation of our work. Our manuscript describes a technically sound piece of scientific research with data that supports the conclusions. Experiments are conducted rigorously, with appropriate controls, replication, and sample sizes. The conclusions are drawn appropriately based on the data presented.

2. Has the statistical analysis been performed appropriately and rigorously?

Reviewer #1: Yes

**Response:**Your affirmation of the significance of our results has been a source of great satisfaction and validation.

Reviewer #2: I Don't Know

**Response:** We sincerely thank reviewer 2 for their important comments. We agree that it is critical to ensure transparency and rigor in statistical analysis. To this end, we have revised the manuscript comprehensively:

In the 'Statistical Processing' section, we provide Multiple statistical comparisons.The part on page 15 , lines 302-309 has been revised as follows:Measurement data were presented as mean ± standard deviation (M ± SD). An independent samples t-test was conducted, and statistical analyses were performed using SPSS version 16. A p-value of less than 0.05 was considered to indicate statistical significance.Multiple statistical comparisons were performed on the primary outcomes (Mid-term exam score,final exam score and overall satisfaction score). To control the increased risk of Type I error (family-wise error rate) associated with multiple comparisons, a Bonferroni correction was applied. The significance level was adjusted to p<0.025 (0.017 / 3)

In the 'Questionnaire Survey' section, we provide Reliability analysis.The part on page 15 , lines 292-301 has been revised as follows:The initial Questionnaire Survey was generated based on a comprehensive literature review of blended learning evaluations and discussions with subject matter experts (experienced pathophysiology teachers), subsequently utilizing Wenjuanxing for data collection and statistical analysis. The questionnaire facilitated an anonymous survey among students in the experimental cohort to ascertain their subjective assessments of the pathophysiological blended teaching approach, which is aligned with the practicing physician qualification examination. This initiative aimed to enhance comprehension of the instructional context and enable the educators to refine their pedagogical strategies accordingly. The Reliability Analysis results that Cronbach's alpha is greater than 0.7 when the questionnaire is available.

In the “Teaching effect”section, we fully report all statistical results, including Cohen’s d= 2.01, 95% CI.The part on page 15-17 has been revised as follows:Table 2. Mid-term examination scores of pathophysiology in the experimental group and the control group and Table 3: Final examination scores of Pathophysiology in the experimental group and the control group

We have updated the relevant graphs to present the statistical comparison results more clearly.

We believe that with these revisions, the statistical analysis process of the manuscript is now clear, reproducible, and meets rigorous scientific standards.

3.Have the authors made all data underlying the findings in their manuscript fully available?
Reviewer #1: Yes

Reviewer #2: Yes

**Response to Reviewers:**We thank both reviewers for confirming that the data underlying our findings are fully available. As stated in the Data Availability Statement, all data are provided within the manuscript and its supplementary information files.

4.Is the manuscript presented in an intelligible fashion and written in standard English?
Reviewer #1: Yes

Reviewer #2: Yes

**Response to Reviewers:**We thank both reviewers for their positive assessment of the manuscript's clarity and English language quality.

5.Review Comments to the Author
Reviewer #1: 1. Provide reference for : In China, the National Medical
Licensing Examination was inaugurated in 1998, with the inaugural clinical examination conducted in September 1999

**Response:**We thank the reviewer for this constructive suggestion. We have now added a supporting reference to this statement. The sentence on page 3 , lines 55-56 has been revised as follows:

"In China, the National Medical Licensing Examination was inaugurated in 1998, with the inaugural clinical examination conducted in September 1999."

Added Reference:

[4]Practicing Physician Law of the People's Republic of China (Order No. 5 of the President of the People's Republic of China). National Health Commission of the People's Republic of China. https://www.nhc.gov.cn/wjw/flfg/199806/b71860019f3949c9b3e73d9bb7f835c5.shtml. Published 1998. Accessed June 26.
2. Authors should place 1. Research object under Research method.

**Response:**We thank the reviewer for this suggestion. We have now moved the "Research object" section to become a subsection under the "Research method" section, as recommended. Please see page 6, lines 119-141.
3.Participants were drawn from the 2022 undergraduate cohorts of Clinical Medicine and Imaging, specifically from classes 1-22 and 117-122, totaling 876 students' - authors may need to mention the participants from only 1 university/college - to provide better clarity for international readers.

**Response:**We appreciate the reviewer's insightful comment. To avoid any ambiguity and to better frame the study's setting for an international readership, we have revised the manuscript to clearly indicate that the study was conducted at a single university. The updated sentence on page 6, lines 120-121 is as follows:"This study included participants from the 2022 undergraduate students of Clinical Medicine and Imaging at *North Henan Medical University*. Students were sampled from classes 1-22 and 117-122, yielding a total sample size of 876."This revision provides a clearer understanding of the study's institutional scope.
4. Authors could provide Section : Literature review - write up on blended learning and traditional learning globally and narrow down to China context

**Response:**We sincerely thank the reviewer for this constructive and crucial suggestion. We agree that a dedicated literature review section will significantly strengthen the introduction and better contextualize our study. Following the reviewer's guidance, we have added the content of "Literature Review on Blended and Traditional Learning" within the introduction (please see pages4-5,line 81-101).In this new section, we have:

First, discussed the global landscape of blended learning versus traditional learning, Then, we specifically narrowed down the focus to the Chinese educational context, reviewing relevant studies and the unique application, challenges, and evolution of blended learning within China's higher medical education system.

This new section provides a comprehensive theoretical foundation and clearly establishes the research gap that our study aims to address, both globally and specifically in China. We believe this addition has greatly improved the manuscript's scholarly depth.

Reviewer #2: The manuscript addresses an important and timely issue in medical education: the effectiveness of blended learning compared to traditional teaching, specifically in preparing students for the National Medical Licensing Examination. The topic is relevant to a global readership, and the study has potential to contribute to the literature on instructional reform in medical education. However, the manuscript requires substantial revisions to meet the methodological and reporting standards expected of PLOS ONE.

**Response:**We sincerely thank the Editor and the Reviewers for their time and valuable comments on our manuscript, We greatly appreciate the positive recognition of the importance and timeliness of our research topic on the effectiveness of blended learning in medical education. We also thank you for the constructive feedback, which has helped us identify key areas for improvement.

We have carefully considered all the points raised and have undertaken a substantial revision of the manuscript to address the concerns regarding methodological rigor and reporting standards.
Major Comments
1. Study Design and Methodology
• The study is described as a comparative analysis, but it is essentially a quasi‑experimental design without randomization. This limitation should be explicitly acknowledged in the Methods and Discussion sections.

**Response:**We thank the reviewer for this insightful comment. We agree that the study design is more accurately described as a quasi-experimental one. We have now revised the Methods section to explicitly label the design as a "quasi-experimental study", the part on page 6 , lines 124-125 has been revised as follows:The study employed a quasi-experimental, non-randomized, comparative design.

We also have added a dedicated discussion of this limitation, including its potential impact on the results, in the Discussion section,the part on page 23-24 , lines 488-500 has been revised as follows:This study possesses several limitations that warrant consideration when interpreting the results. Primarily, the quasi-experimental design, although practical, does not eliminate the potential impact of unmeasured confounding variables. Despite controlling for admission scores and key demographic factors, other elements, such as subtle variations in instructor enthusiasm and teaching effectiveness between the two groups, or differences in students' prior knowledge and self-directed learning skills not captured by admission scores, may have influenced the outcomes. Additionally, the generalizability of our findings is potentially constrained. The research was conducted at a single medical college and exclusively addressed the subject of pathophysiology. Consequently, the results may not be directly applicable to other institutions with different student populations and educational cultures, or to other medical subjects with distinct pedagogical requirements. Future multi-institutional, multi-disciplinary randomized controlled trials are necessary to validate these findings and enhance their external validity.

• The allocation of classes to experimental and control groups appears based on convenience (first four vs. last four classes). This introduces potential selection bias. Please clarify how baseline equivalence was ensured beyond admission scores.

**Response:**We thank the reviewer for raising this critical point regarding potential selection bias. We acknowledge that using the first four and last four classes is a limitation of our quasi-experimental design.However, I still want to explain the situation to you. According to the curriculum arrangement requirements of the educational administration system of the researcher's school, the study has to be divided in this way, and the author cannot intervene. What the author wants to illustrate that every student is extremely creative and subjective. Despite the lack of a thorough randomization design, the results are still instructive and important.

• The description of the intervention (blended teaching) is detailed, but the control condition is less thoroughly described. For transparency, provide equal detail on the control group’s teaching methods.

**Response:**We thank the reviewer for this valuable suggestion to enhance the transparency of our manuscript. We agree that a detailed description of the control condition is essential for a clear understanding of the comparison. We have now revised the Methods section to provide a comprehensive and parallel description of the teaching methods used in the control group, matching the level of detail provided for the intervention group. The additions include specific instructional resource use, instructional strategies, and control conditions for teacher participation.The part on page 10 , lines 185-196 and page13, lines 258-268 has been revised as follows:

“The control cohort engaged in a traditional lecture-based teaching approach, specifically aligned with the examination framework of the Practicing Physician Qualification Examination. Instructors utilized PowerPoint presentations to deliver a comprehensive and systematic coverage of the syllabus content. Instruction was delivered in a conventional classroom setting, where students primarily assumed a passive role, focusing on listening to lectures and taking notes. Classroom interaction was predominantly initiated by instructors posing questions, to which students responded collectively or individually, sometimes facilitated by the Rain Classroom platform. The course included practice questions and answers as part of the curriculum. Students primarily relied on assigned textbooks and lecture notes provided by instructors as their main learning resources. The online learning platform was used solely for downloading course materials and did not include any obligatory online activities or discussions.”

“1.3.4 Classroom arrangement for the control group

Preparation for Class in the Control Group: Teachers collaboratively prepare PowerPoint presentations and lesson plans. Additionally, they utilize the Rain Classroom platform to introduce new chapters.

Implementation in Class of the control group:The classroom dynamics are predominantly influenced by the teacher's instructional approach. Interaction between students and the teacher primarily consists of the teacher posing questions, to which students respond collectively, or individual students are selected to provide answers.

Review after Class of the control group:There will be practice questions in the class, and at the end of the class, there will be a few thinking questions, which are mainly from the textbook and have answers.”

2. Ethics and Approvals
• The manuscript currently states “N/A” for ethics approval. Given that students were surveyed and their academic performance analyzed, institutional ethics approval (or a waiver) is typically required. Please clarify whether approval was obtained, and if not, justify why it was deemed unnecessary.

**Response:**We thank the reviewer for this important question regarding research ethics. This study involved the analysis of anonymized academic performance data and anonymized survey responses that were collected as part of a routine educational evaluation of the teaching methods. No personal identifying information was accessed or analyzed.We need to explain to you the issue regarding the use of student grades to obtain their informed consent. In China, after each exam, schools require teachers to conduct a grade analysis based on the students' scores. This grade analysis is then shared with the students, and Chinese students are informed.In the Chinese education system, analyzing students' grades to improve teaching quality is a very common practice. Chinese students are informed of this from the very beginning of their schooling.
3. Statistical Analysis
• The statistical methods are limited to independent t‑tests. Given the large sample size (n=876), effect sizes (e.g., Cohen’s d) should be reported to contextualize the practical significance of findings.

**Response:**We thank the reviewer for this excellent suggestion, which strengthens the interpretation of our findings. We agree that reporting effect sizes is crucial, especially with a large sample size, to distinguish statistical significance from practical significance.In response, we have now calculated and reported Cohen's dfor all key continuous outcome comparisons (e.g. Mid-term examination，final exam scores) alongside the results of the independent samples t-tests. The values have been added to the Results section in the text and to Mid-term examination scores of pathophysiology in the experimental group and the control group and Final examination scores of Pathophysiology in the experimental group and the control group. Furthermore, we have added an interpretation of these effect sizes (e.g., small, medium, or large based on conventional thresholds) in the Discussion section to contextualize the practical importance of the observed differences.The part on page 16-17 has been revised as follows:

“Table 2. Mid-term examination scores of pathophysiology in the experimental group and the control group”

“Table 3: Final examination scores of Pathophysiology in the experimental group and the control group”

• Multiple outcomes (mid‑term, final, questionnaire responses) are analyzed, but no correction for multiple comparisons is mentioned. Please address this.

**Response:**We thank the reviewer for highlighting this important methodological point. We acknowledge that the analysis of multiple outcomes increases the family-wise error rate (FWER), and we agree that this should be addressed.In response to this comment, we have applied the Bonferroni correction to adjust the significance level for our key family of tests. Given that our primary outcomes were the Mid-term exam score, the final exam score and the total score on the learning satisfaction questionnaire, we considered these three tests as a family. The adjusted significance level is therefore α = 0.05 / 3 = 0.017. We have updated the Results section to report p-values against this new threshold and have clarified in the Methods section that a Bonferroni correction was applied to control the FWER for the primary outcomes.We have added a sentence in the Statistical Analysis subsection of the Methods to explicitly state this approach.The part on page 15,line 301-310 has been revised as follows:

“Multiple statistical comparisons were performed on the primary outcomes (Mid-term exam score,final exam score and overall satisfaction score). To control the increased risk of Type I error (family-wise error rate) associated with multiple comparisons, a Bonferroni correction was applied. The significance level was adjusted to p<0.025 (0.017 / 3)”
• The reporting of p‑values is inconsistent (e.g., “0.046 5”). Ensure correct formatting and precision.

**Response:**We thank the reviewer for pointing out the inconsistency in p-value reporting. We have carefully reviewed the entire manuscript and standardized the format of all p-values according to conventional academic practice. They are now reported to three decimal places, with values below 0.001 denoted as p<0 .001.
4. Results Presentation
• Tables are not formatted according to journal standards. For example, Table 2 is fragmented in the draft. Please revise for clarity and consistency.

**Response:**We thank the reviewer for this feedback on the presentation of our tables. We have carefully reviewed the "Instructions for Authors" for the journal and have reformatted all tables (especially Table 2) to comply fully with the journal's stylistic standards. This includes adjusting the table structure, font, and borders to prevent fragmentation and ensure clarity. The revised tables are now clear, consistent, and integrated into the manuscript without awkward page breaks.

The part on page 16-17 has been revised as follows:

| Mid-term performance | Variable | | n | M±SD | t | p | Cohen’s d | 95% CI |
| --- | --- | --- | --- | --- | --- | --- | --- | --- |
|  | Teaching methods | Experimental group | 445 | 66±1.708 | 2.501 | 0.047 | 2.01 | 0.218,3.806 |
|  |  | Control group | 431 | 62.7±2.217 |  |  |  |  |

**Table 2. Mid-term examination scores of pathophysiology in the experimental group and the control group**

| Final performance | Variable | | n | M±SD | t | p | Cohen’s d | 95% CI |
| --- | --- | --- | --- | --- | --- | --- | --- | --- |
|  | Teaching methods | Experimental group | 444 | 77±1.155 | 3.667 | 0.011 | 2.894 | 0.821, 4.973 |
|  |  | Control group | 431 | 74.25±0.96 |  |  |  |  |

**Table 3: Final examination scores of Pathophysiology in the experimental group and the control group**

• The questionnaire results are summarized descriptively (percentages), but no validated instrument is cited. Was the survey piloted or validated? This should be clarified.

**Response:**We thank the reviewer for this important question regarding the validity of our survey instrument. The questionnaire used in this study was developed by the research team specifically for this teaching intervention, as no existing validated instrument fully captured the dimensions of student engagement and satisfaction we aimed to measure.

To address the reviewer's concern about validation, we would like to clarify the steps we took to ensure the instrument's robustness:

Item Generation: The initial item pool was generated based on a comprehensive literature review of blended learning evaluations and discussions with subject matter experts (experienced pathophysiology teachers).

Pilot Testing: The draft questionnaire was piloted with a small group of students (n=30) from a parallel cohort not involved in the main study. This helped assess the clarity, comprehensibility, and time required to complete the survey.

Face and Content Validity: The pilot feedback and expert reviews were used to refine the items for clarity and relevance, thereby establishing face and content validity.

Reliability Analysis: In the main study, we assessed the internal consistency reliability of the key satisfaction scale using Cronbach's alpha, which was found to be 0.725, indicating acceptability(Table 4 on page 19).

We have now added a detailed description of the questionnaire's development, piloting, and validation process in the Methods section .

4. Discussion and Interpretation
   • The discussion tends to overstate the causal impact of blended learning. Given the quasi‑experimental design, conclusions should be tempered to emphasize association rather than causation.

**Response:**We thank the reviewer for this crucial comment regarding causal language. We fully agree that given the quasi-experimental design of our study, it is important to avoid overstating causal inferences.In response, we have carefully revised the entire Discussion and Conclusion sections to temper our language. We have replaced definitive causal claims with phrasing that emphasizes the associationbetween the blended learning intervention and the observed outcomes, and we have framed the findings as suggestinga potential benefit rather than proving it. We have also strengthened the section on limitations to more explicitly acknowledge that the non-randomized design prevents firm causal conclusions.Like the page 10, line 394-395:Blended classroom teaching was associated withstudents' academic performance.

• The manuscript would benefit from situating findings within the broader international literature on blended learning in medical education (e.g., systematic reviews, meta‑analyses).

**Response:**We thank the reviewer for this valuable suggestion to enhance the scholarly context of our work. We agree that situating our findings within the broader international literature will strengthen the discussion.In response, we have thoroughly reviewed the recent high-impact systematic reviews and meta-analyses on blended learning in medical education. We have now integrated these key references into the Introduction and the Discussion section. Specifically, In the "Introduction" section of the paper,we have strengthened and updated the literature review, specifically citing high-quality systematic reviews and meta-analyses published in recent years (e.g., work by Vallée A et al., 2020 and Li M et al., 2025 ) to draw a clearer picture of the current field and key unresolved issues, thereby providing a more solid theoretical foundation for our study.

More importantly, in the "Discussion" section, we have significantly revised and expanded. We contrasted our findings in detail with the main conclusions of the systematic review described above. Specifically:We point to the consistency of this study with the mainstream literature in terms of referring to a specific finding, e.g., the Internet into traditional educational methodologies, prompting innovative teaching reforms. The utilization of online tools has substantially enhanced both the efficiency of instruction and the convenience of student learning. As for the Discussion of this study The utilization of mind maps and knowledge graphs facilitates a more intuitive comprehension of the examination's key elements for students, which was complementary with some literatures.

• Limitations are acknowledged but should be expanded to include issues of generalizability (single institution, single subject area) and potential confounders (teacher effects, prior student ability).

**Response:**We thank the reviewer for this insightful comment that helps us present a more balanced and critical view of our study's limitations. We have expanded the 'Limitations' section in the Discussion to explicitly address the points raised.We have now added detailed discussions regarding: 1) the limited generalizability of our findings due to the single-institution, single-subject design, and 2) the potential confounding effects of unmeasured variables, specifically differences in instructor effectiveness and prior student ability beyond the admission scores we controlled for. We believe these additions significantly strengthen the manuscript's rigor.
6. Data Availability
• The current statement (“Some of the data… original questionnaire in supplementary materials”) does not meet PLOS ONE’s open data policy. Authors must provide either a public repository link or a clear justification for restrictions.
**Response:**Thank you for your valuable comment. We have revised the data availability statement in accordance with PLOS ONE's open data policy.Midterm Scores, Final Scores, and Average Scores in “S4 File” Research data is File4 Title. Grading of Test Difficulty Coefficients in S5 File. The original questionnaire data have been deposited in “S6 File.Original questionnaire is File6 Title.”This information has been updated in the manuscript.

Minor Comments
• The English language requires editing for grammar and conciseness. For example, phrases such as “the adaptability of 22nd grade clinical medicine major students” should be revised for clarity.

**Response:** We sincerely thank the reviewer for this valuable feedback. We apologize for any language errors that affected the clarity of our manuscript. We have thoroughly revised the entire manuscript to improve the grammar, conciseness, and overall readability.Specific changes made in response to this comment include:

The example phrase has been revised to: “the adaptability of the Grade 22 clinical medicine students” to accurately reflect the educational stage of the participants.

The entire manuscript has been carefully edited to eliminate grammatical errors, reduce wordiness, and enhance clarity. We have paid particular attention to replacing ambiguous or redundant phrases with more direct and academic expressions.

To ensure the highest quality, the language of the manuscript has been polished by a professional editing service with expertise in medical writing.

We believe the manuscript now meets the journal's standard for English language quality. All changes have been implemented throughout the text.
• The abstract should include specific statistical results (means, SDs, p‑values) rather than general statements.

**Response:**We thank the reviewer for this excellent suggestion. We agree that including specific statistical data will make the abstract more informative and compelling. We have now thoroughly revised the abstract to replace all general statements with precise statistical results.Key changes made to the abstract include:

We replaced the general statement about exam scores with specific between-group comparisons: "Students in the blended learning group scored significantly higher on the mid-term examination (M = 66, SD = 1.708) than those in the traditional lecture group (62.7,SD =2.217), t(876) = 2.501, p＝0.047, Cohen’s d= 2.01, 95% CI [0.218, 3.806]. "

We quantified student satisfaction using mean and standard deviation from the Likert scale data: "Students in the blended learning group scored significantly higher on the final examination (M = 77, SD = 1.155) than those in the traditional lecture group (74.25,SD =0.96), t(876) = 3.667, p＝0.011, Cohen’s d= 2.894, 95% CI [0.821, 4.973]"

We believe the revised abstract now provides a clear, data-driven summary of our key findings, allowing readers to better assess the strength and significance of our results. These changes have been made in the manuscript.
• Figures (e.g., instructional design diagrams) should be redrawn for clarity and labeled according to journal style.

**Response:**We thank the reviewer for the constructive feedback. All figures (including instructional design diagrams) have been redrawn to improve clarity and comply with PLOS ONE's style requirements. Specifically:

Vector-based tools (Microsoft PowerPoint) were used to enhance line sharpness and text legibility.

Labels, axis titles, and legends were standardized to Arial 12 pt font.

Redundant elements were removed to simplify visual interpretation.The updated figures are now uploaded as PDF (*.pdf) in the submission system.
• References: Ensure consistency in formatting (some Chinese references lack full details in English).

**Response:** We sincerely thank the reviewer for pointing out this oversight. We have now carefully reviewed and reformatted the entire reference list to ensure complete consistency according to the journal's required style.The specific actions taken are as follows:

Translation of Chinese References: All Chinese references have been provided with their complete and official English translations. This includes the author names (in pinyin), the translated article title within square brackets, and the official English name of the journal.

Uniform Formatting Check: We have meticulously checked every reference for consistency in author naming, journal title presentation (full name), punctuation, and the inclusion of DOIs where available.

For example, the Chinese reference has been updated from:Zhang Chunqing, Wang He, Song Fangting, et al. Thoughts on the Clinical Medical Education Model in China [J]. China Higher Medical Education,2023,01: 22-23.

To the corrected format:Zhang CQ,Wang L,Song FT,et al.Thinking about the clinical medical education model in China[J].China Higher Medical Education,2023,01: 22-23.

We believe the reference list now fully meets the journal's formatting standards. All changes are reflected in the manuscript.
