# Supplementary material for: A comparative analysis of academic outcomes in blended versus traditional instructional approaches: An examination within the context of the National Medical Licensing Examination
Source: PLoS One. 2026 Apr 17;21(4):e0346793. doi: 10.1371/journal.pone.0346793 (PMC13089738; doi:10.1371/journal.pone.0346793)
Supplement: Attachment — Submitted filename: Response to Reviewers3.20.docx [file pone.0346793.s010.docx]

Response Letter

Manuscript Number:PONE-D-25-34517.

Title:***A comparative analysis of academic outcomes in blended versus traditional instructional approaches: An examination within the context of the National Medical Licensing Examination.***

Mar. 20th, 2026

Dear Editor Mukhtiar Baig, Ph.D., devi and Reviewers:

On behalf of my co-authors, we thank you for giving us a chance to revise and improve the quality of our article.

We have read the reviewers' and your comments carefully and have made revision which marked in red in the paper. We have tried our best to revise our manuscript according to the comments: “A Comparative Analysis of Academic Outcomes in Blended versus Traditional Instructional Approaches: An Examination within the Context of the National Medical Licensing Examination. (PONE-D-25-34517)”.

Attached please find the revised version, which we hope that you will find this updated manuscript to your satisfaction and consider it for publication as an article in *PLOS ONE*. Here is a point-by-point response to the reviewers' comments and concerns.

Thank you for taking the time to consider our research and we look forward to hearing from you at your earliest convenience.

Sincerely,

Siyu Zhang

North Henan Medical University,

Xinxiang, HeNan China

E－mail:shiyu15937135473@163.com

1. If the authors have adequately addressed your comments raised in a previous round of review and you feel that this manuscript is now acceptable for publication, you may indicate that here to bypass the “Comments to the Author” section, enter your conflict of interest statement in the “Confidential to Editor” section, and submit your "Accept" recommendation.

Reviewer #1: All comments have been addressed

**Response:**We are pleased that the reviewer confirms all previous comments have been addressed. Thank you for your thorough and constructive feedback throughout the review process, which has significantly strengthened our manuscript.

Reviewer #2: All comments have been addressed

**Response:**We appreciate the reviewer's confirmation that all concerns raised in the previous round have been satisfactorily addressed. Your insightful comments have been invaluable in improving our work.

2. Is the manuscript technically sound, and do the data support the conclusions?

The manuscript must describe a technically sound piece of scientific research with data that supports the conclusions. Experiments must have been conducted rigorously, with appropriate controls, replication, and sample sizes. The conclusions must be drawn appropriately based on the data presented.

Reviewer #1: Yes

**Response:**We thank the reviewer for confirming that the manuscript is technically sound and that the data support the conclusions. We appreciate this positive assessment of our work.

Reviewer #2: Yes

**Response:**We are grateful to the reviewer for acknowledging that the experiments were conducted rigorously and that the conclusions are appropriately drawn from the data presented. Thank you for your valuable time and expertise in reviewing our manuscript.

3. Has the statistical analysis been performed appropriately and rigorously?

Reviewer #1: Yes

**Response:**We thank the reviewer for confirming that the statistical analysis in our manuscript has been performed appropriately and rigorously. We appreciate this positive assessment of our methodological approach.

Reviewer #2: Yes

**Response:**We are grateful to the reviewer for acknowledging the appropriateness and rigor of our statistical analysis. Your validation of our statistical methods is highly valued.

4. Have the authors made all data underlying the findings in their manuscript fully available?

The [PLOS Data policy](http://www.plosone.org/static/policies.action#sharing) requires authors to make all data underlying the findings described in their manuscript fully available without restriction, with rare exception (please refer to the Data Availability Statement in the manuscript PDF file). The data should be provided as part of the manuscript or its supporting information, or deposited to a public repository. For example, in addition to summary statistics, the data points behind means, medians and variance measures should be available. If there are restrictions on publicly sharing data—e.g. participant privacy or use of data from a third party—those must be specified.

Reviewer #1: Yes

**Response:**We thank the reviewer for confirming that all data underlying the findings in our manuscript have been made fully available in accordance with the journal's data policy. We appreciate this verification.

Reviewer #2: Yes

**Response:**We are grateful to the reviewer for acknowledging that our data availability meets the required standards. As stated in our Data Availability Statement, all relevant data are （e.g., provided in the Supporting Information files with accession number S4_File，S5_File）.

5. Is the manuscript presented in an intelligible fashion and written in standard English?

PLOS ONE does not copyedit accepted manuscripts, so the language in submitted articles must be clear, correct, and unambiguous. Any typographical or grammatical errors should be corrected at revision, so please note any specific errors here.

Reviewer #1: Yes

**Response:**We thank the reviewer for confirming that our manuscript is presented in an intelligible fashion and written in standard English. We appreciate this positive assessment of the language quality.

Reviewer #2: Yes

**Response:**We are grateful to the reviewer for acknowledging that the language in our manuscript is clear, correct, and unambiguous. Your validation of the writing quality is highly valued.

6. Review Comments to the Author

Please use the space provided to explain your answers to the questions above. You may also include additional comments for the author, including concerns about dual publication, research ethics, or publication ethics. (Please upload your review as an attachment if it exceeds 20,000 characters)

Reviewer #1: 1. Authors need to remove data / numbers in abstract section , just provide summary of findings

2. Authors could proofread the manuscript.

**Response:**We thank the reviewer for the valuable comments and suggestions.

Comment 1: "Authors need to remove data / numbers in abstract section, just provide summary of findings"

Response: Thank you for this suggestion. We would like to clarify a situation. During the first revision, another reviewer suggested adding the data to the abstract. After discussing with the team, we have summarized the data and presented the corresponding conclusions.. The revised abstract now reads:

Objective: This research examines the distinctions between the blended teaching approach and the traditional instructional method within the context of pathophysiology, utilizing the Practicing Physician Qualification Examination as a foundational framework. The objective is to elucidate the effects of the blended teaching model and to provide insights and recommendations from diverse perspectives to guide future reforms in medical education. Method: The research methodology comprised a comparative analysis of students' academic performance within the institution, supplemented by the administration of a questionnaire survey. The experimental group participated in a blended classroom teaching model designed to align with the content of the Practicing Physician Examination, while the control group adhered to a conventional teaching model based on the identical examination content. Result: The students in the blended learning group demonstrated significantly higher performance on both the mid-term and final examinations compared to those in the traditional lecture group. The instructional method showed a substantial impact on student achievement. Additionally, a majority of students in the experimental group perceived that the blended classroom teaching model, designed around the Practicing Physician Examination, enhanced their competence for the examination.Conclusion: The adoption of a blended classroom teaching model, focused on the Practicing Physician Examination, has significantly enhanced educational outcomes in pathophysiology and substantially improved students' preparedness for the Practicing Physician Examination.

Comment 2: "Authors could proofread the manuscript"

Response: Thank you for this suggestion. We have carefully proofread the entire manuscript to correct any typographical or grammatical errors.Eg:The sentence on page 1 , lines 5 “Baosheng Yan” has been revised as follows:Baosheng Yang.

Reviewer #2: Accept the paper. It is an important topic for the publication in the plos one and I wish the authors well.

**Response:**We sincerely thank the reviewer for the positive assessment and for recommending acceptance of our manuscript. We are pleased that you find our topic important and suitable for publication in PLOS ONE. Your encouraging words are greatly appreciated.
